# Genomic and Acoustic Biogeography of the Iconic Sulphur-crested Cockatoo Clarifies Species Limits and Patterns of Intraspecific Diversity

**DOI:** 10.1093/molbev/msae222

**Published:** 2024-10-24

**Authors:** Arthur F Sands, Astrid A L Andersson, Kerry Reid, Taylor Hains, Leo Joseph, Alex Drew, Ian J Mason, Frank E Rheindt, Caroline Dingle, Juha Merilä

**Affiliations:** Area of Ecology and Biodiversity, School of Biological Sciences, The University of Hong Kong, Hong Kong, Hong Kong SAR; Area of Ecology and Biodiversity, School of Biological Sciences, The University of Hong Kong, Hong Kong, Hong Kong SAR; Area of Ecology and Biodiversity, School of Biological Sciences, The University of Hong Kong, Hong Kong, Hong Kong SAR; Committee on Evolutionary Biology, University of Chicago, Chicago, IL, USA; Negaunee Integrative Research Center, Field Museum of Natural History, Chicago, IL, USA; Australian National Wildlife Collection, CSIRO National Research Collections Australia, Canberra, Australia; Australian National Wildlife Collection, CSIRO National Research Collections Australia, Canberra, Australia; Australian National Wildlife Collection, CSIRO National Research Collections Australia, Canberra, Australia; Department of Biological Sciences, National University of Singapore, Kent Ridge, Singapore; Area of Ecology and Biodiversity, School of Biological Sciences, The University of Hong Kong, Hong Kong, Hong Kong SAR; Biology Department, Capilano University, North Vancouver, BC, Canada; Area of Ecology and Biodiversity, School of Biological Sciences, The University of Hong Kong, Hong Kong, Hong Kong SAR; Ecological Genetics Research Unit, Faculty of Biological and Environmental Sciences, University of Helsinki, Helsinki, Finland

**Keywords:** Australia, *Cacatua galerita*, conservation, New Guinea, phylogenomics, phylogeography, systematics

## Abstract

Many highly recognizable species lack genetic data important for conservation due to neglect over their hyperabundance. This likely applies to the Sulfur-crested Cockatoo (*Cacatua galerita*), one of the world's most iconic parrots. The species is native to Australia, New Guinea, and some surrounding Melanesian islands of the latter. Four subspecies are currently recognised based on morphology. Australian subspecies and populations are abundant, but several factors threaten those in New Guinea and Melanesia. Genetic data from natural populations are scarce—information that is vital to identifying evolutionarily significant units (ESUs) important for modern conservation planning. We used whole-genome resequencing to investigate patterns of differentiation, evolutionary affinities, and demographic history across *C. galerita*'s distribution range to assess whether currently recognised subspecies represent ESUs. We complement this with an assessment of bioacoustic variation across the species' distribution landscape. Our results point to *C. galerita sensu lato* (*s.l.*) comprising two species. We restrict *C. galerita sensu stricto (s.s.)* to populations in Australia and the Trans-Fly ecodomain of southern New Guinea. The second species, recognised here as *Cacatua triton*, likely occurs over much of the rest of New Guinea. Restricting further discussion of intraspecific diversity in *C. triton*, we show that within *C. galerita s.s.* two ESUs exist, which align to *Cacatua galerita galerita* in eastern Australia and southern New Guinea and *Cacatua galerita fitzroyi* in northern and north-western Australia. We suggest that the evolution of these species and ESUs are linked to Middle and Late Pleistocene glacial cycles and their effects on sea level and preferential habitats. We argue that conservation assessments need updating, protection of preferential forest and woodland habitats are important and reintroductions require careful management to avoid possible negative hybridization effects of non-complementary lineages.

## Introduction

Australasian Cacatuidae (cockatoos) have become symbolic of Australia, which is famous for its many unique lineages that evolved over an extended evolutionary period. Among most recognizable of all, the Sulfur-crested Cockatoo, *Cacatua galerita* (Latham 1790), is undoubtedly iconic (Cameron 2007) and has appeared on currency, postage stamps, and other symbols of national and local importance.

While many bird species are under threat in Australia, *Cacatua galerita* is abundant and may only be in marginal decline (Cameron 2007; Mulawka 2014). Small expansions in the species' distribution range and abundance are still observed in some places (Burgin and Saunders 2007; Scroggie and Ramsey 2021). Moreover, the existence of large flocks, which can have devastating effects on agriculture and negative interactions with humans in urban environments (Klump et al. 2021), means the species is often considered a pest in some areas (Ackroyd 1989; Cameron 2007). The establishment of a growing number of protected zones in Australia, the country's ratification of CITES (with the species being listed on “Appendix II” from 1981; checklist.cites.org) and the implementation of national laws controlling hunting and export/import of Australian parrots in their native range (Ackroyd 1989), as well as *C. galerita*'s ability to adapt to urban and rural Australian settings (Aplin et al. 2021; Klump et al. 2021; Fehlmann et al. 2024), have probably helped its survival prospects.

**Fig. 1.**
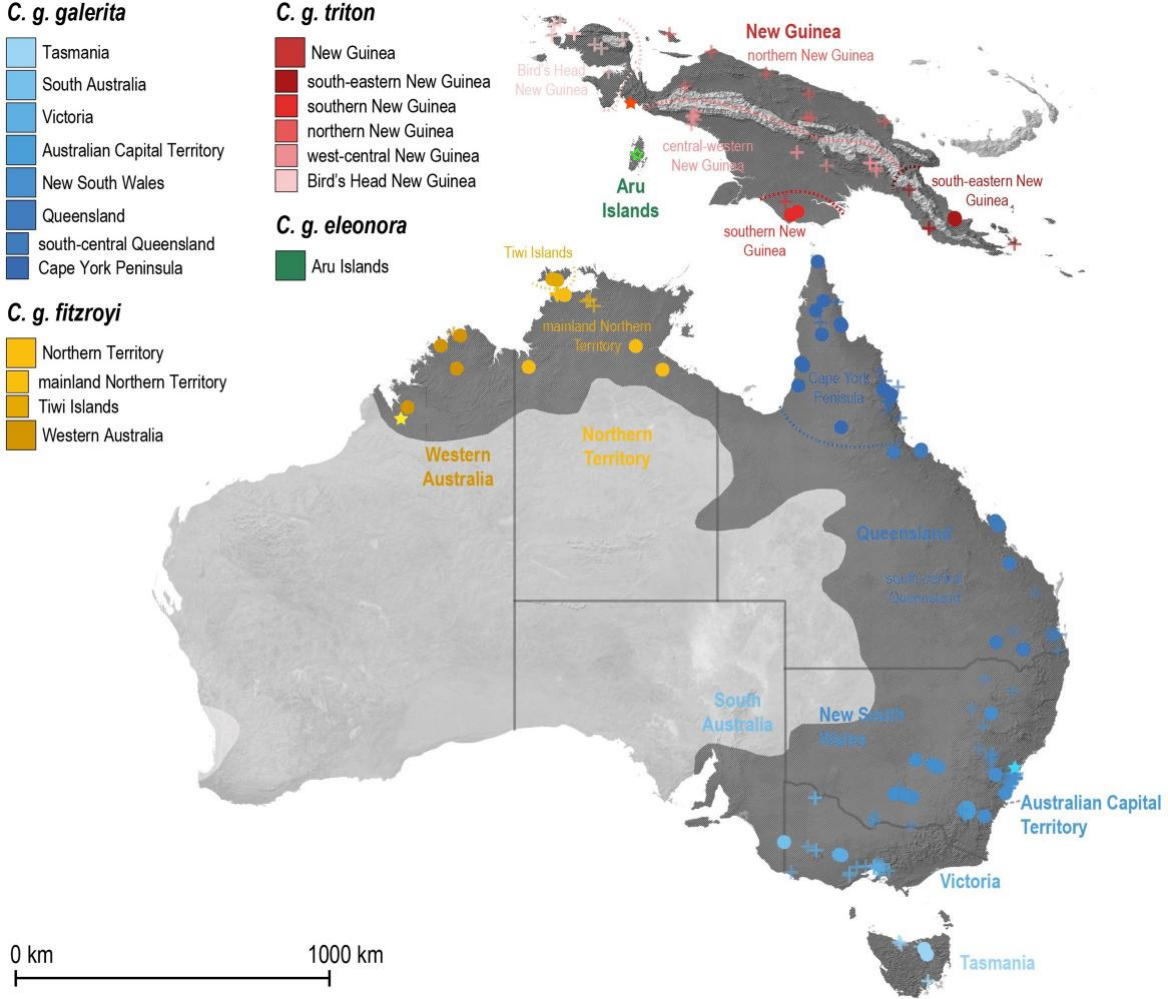
Sampling localities with traditional (morphologically diagnosed) subspecies assignments of *C. galerita* across the species' native (black shading) and introduced (light grey shading) distribution ranges. Locations are shown of samples used in genomic (coloured dots) and bioacoustic (coloured crosses) analyses and their colours are coded per the key. Brightly coloured stars indicate the type localities of taxa; that for *C. g. eleonora* is only bordered in colour to indicate the type locality's imprecision.

Like many other taxa that have become linked to Australian identity (e.g. wallabies, echidnas, and birds such as kookaburras), *C. galerita* also occurs naturally beyond Australia in the larger Australasian biogeographic realm (Cameron 2007; BirdLife International 2023). Its distribution includes Melanesia (i.e. New Guinea, the Aru Islands, and smaller associated satellite islands of the former; Fig. 1). The iconic link to Australia and the abundance and conservation of the species there (Ackroyd 1989) could mislead perceptions of the plight of the species in Melanesia, where *C. galerita* faces increasing challenges to its survival (Cameron 2007; Mulawka 2014; Olah et al. 2016). For example, habitat loss and fragmentation (through deforestation and land-use change; Filer et al. 2009; Sloan et al. 2019; Gaveau et al. 2021), poaching for the illegal pet trade and hunting for traditional customs (Smiet 1982; Paddock 2002; Pangau-Adam and Noske 2010), erratic weather patterns (associated with climate change; Şekercioğlu et al. 2012) and a lack of suitable conservation resources and governance (Smiet 1982; Blazey 2012; Sloan et al. 2019) are all thought to be contributing to population declines and increasing scarcity in abundance, driving a growing need for conservation action (Cameron 2007; Mulawka 2014; Olah et al. 2016).

For most species, effective conservation management increasingly relies upon identifying the genetic structure within species to inform the protection of genetic variation and the mechanisms that allow species to continuously evolve and adapt to their surroundings. In this regard, protecting and preserving unique intraspecific lineages or evolutionarily significant (or stable) units (ESUs; Moritz 1994) within a species across its range are seen as important to its long-term success and survival (Des Roches et al. 2018; Allendorf et al. 2022). The loss of genetic diversity or intraspecific ESUs that have co-evolved with specific environmental settings can lead to a species becoming more vulnerable to extinction. Further, ecosystem imbalances may arise if a species is lost or replaced locally by other species or even other ESUs of the same species (Des Roches et al. 2018). Assessments of the genetic diversity, demography, and recent evolutionary history of an ESU can also direct tailored conservation actions that are suitable to addressing specific concerns.

Avian subspecies, described using traditional morphological, ecological, and geographic approaches, often translate into valid intraspecific ESUs due to correlated patterns of genetic diversity and traditional data frequently reflecting independent evolutionary histories (e.g. Jønsson et al. 2019; Rodríguez et al. 2020; Dalton et al. 2022; Wu et al. 2022). However, a lack of knowledge on intraspecific variation and local extinction events in areas of former occurrence or where two subspecies have a geographical zone of intergradation can occasionally complicate the use of subspecies as ESUs for conservation (e.g. Jønsson et al. 2019; Sadanandan et al. 2020; Davis et al. 2021; Sánchez-Nivicela et al. 2021). For example, taxonomic inflation can occur when morphological differences do not correspond to genetic differentiation, thus straining conservation resources (e.g. Andersson et al. 2024). Vice versa, it is also possible that subspecies described using traditional data can sometimes fail to reflect cryptic diversity (i.e. phenotypically similar lineages which differ in evolutionary histories; Garg et al. 2016; Cahill et al. 2024), therefore excluding some ESUs from protection (e.g. Sánchez-Nivicela et al. 2021; Wu and Rheindt 2022; Cahill et al. 2024).

Little is known of the genetic diversity in *C. galerita* and whether the four currently recognised subspecies represent valid intraspecific ESUs. Between the 1790s and the 1920s, over a dozen species or subspecies were described under varied taxonomic treatments and linkages to *C. galerita* (supplementary table S1, Appendix B, Supplementary Material online). Essentially following morphometric analyses of Mayr (1937) and Mees (1972) for New Guinean populations and Forshaw (1968) for Australian populations, four morphologically diagnosed subspecies are currently recognised (e.g. Dickinson and Remsen 2013; Beehler and Pratt 2016; Clements et al. 2023; Gill et al. 2024): *Cacatua galerita galerita* (Latham 1790), occurring across eastern Australia from Tasmania to the Cape York Peninsula of Queensland; *Cacatua galerita fitzroyi* (Mathews 1912), ranging across northern and north-western Australia (i.e. in the Northern Territory and Western Australia); *Cacatua galerita triton* Temminck 1849, currently ascribed to all mainland New Guinean populations and several associated north-western and south-eastern satellite islands; and *Cacatua galerita eleonora* Finsch 1863, assigned to populations in the Aru Islands. However, an updated revision that encompasses the entire species' native distribution range is needed to assess geographic variation and reappraise diagnostic characters of subspecies warranting recognition. Many early works relied on simple descriptions based on outdated methodology, comparing limited samples or using museum skins, which are possibly subject to some disfigurement over time. Schliebusch and Schliebusch (2001) offered a preliminary genetic perspective and a taxonomic revision of *C. galerita* and close relatives. However, their study relied heavily on just a few molecular markers and primarily birds sourced from aviculture (and so of questionable provenance and genetic integrity).

A validation of the four subspecies as ESUs, based on a phylogenomic approach of wild populations, is needed to assist the planning of management and conservation strategies. Genetic information for wild *C. galerita* is extremely scarce (Provost et al. 2018) and where genetic data exist at all for *C. galerita*, beyond Schliebusch and Schliebusch (2001), it is largely linked to broader phylogenetic-based studies on Cacatuidae and Psittaciformes (e.g. White et al. 2011; Selvatti et al. 2022; Smith et al. 2023), the barcoding of captive birds for rehabilitation back into the wild (e.g. Zein et al. 2017) or zoological gardens' breeding programmes (e.g. Kim et al. 2020). Here, we aim to provide a comprehensive assessment of the intraspecific diversity within *C. galerita* using genomics and bioacoustics and to derive conservation-oriented recommendations. Specifically, we: (i) Test whether there is genomic evidence to support the four currently recognised subspecies as distinct ESUs, (ii) assess for unrecognised cryptic diversity or previously recognised subspecies that need to be reinstated or assigned ESU status, and (iii) bring biogeographic and demographic perspectives to evolutionary events within and between *C. galerita* ESUs to illuminate historical trends and current patterns important for developing sound conservation strategies. To do this, we first generate phylogenies to assess the relationships between taxonomic units and the monophyly of *C. galerita* within *Cacatua*, as well as to assess whether any cryptic interspecific diversity is present. Thereafter, we specifically target intraspecific diversity to assess the structure within *C. galerita*. We also look at the demographic history to detect historical responses to environmental changes, pointing to drivers of population change useful to conservation planning, using mutation rates and generation times as scaling parameters in timeline reconstruction. These inferences are also used to reconstruct a plausible evolutionary and biogeographic history of the species as a whole and validate subspecies which are important to conservation by delimiting ESUs. While traditionally ESUs have been delimited by reciprocal monophyly of mtDNA signatures (Moritz 1994), we delimit ESUs through four tree-based delimitation models. We look for consensus among these delimitation models, while additionally integrating and considering support of reciprocal monophyly in whole-genome-based phylogenies, levels of admixture and genetic distance in justifications. Finally, we analyse vocalizations to test whether genomic patterns are concordant with bioacoustic signals. This bioacoustic assessment provides additional support for assessing the monophyly of *C. galerita* or any cryptic diversity within it and bestows greater resolution for distribution range assessments. Apart from enabling robust insights into the diversity and complex evolutionary history of *C. galerita sensu lato* (*s.l.*), we fashion our results to provide important guidance for management and conservation priorities of this iconic taxon.

## Materials and Methods

### Sample Collection and Laboratory Processing

To build a comprehensive dataset for analyses, we obtained tissue samples of 95 *C. galerita* specimens from the Australian National Wildlife Collection (ANWC; Canberra). Samples represented specimens from across the species' Australian distribution range and two parts of Papua New Guinea (from the South and South–East, respectively), as collected or provided to the ANWC between 1960 and 2020 (Fig. 1; supplementary table S2, Appendix B, Supplementary Material online). As outgroups or to root downstream phylogenomic analyses, our dataset was further supplemented with blood or tissue samples taken from two individuals of both *Cacatua alba* (Müller 1776) (of captive origin, Hong Kong) and *Cacatua sulphurea* (Gmelin 1788) (of feral origin, Hong Kong) and a single *Cacatua moluccensis* (Gmelin 1788) (of captive origin, Hong Kong; supplementary table S2, Appendix B, Supplementary Material online). DNA was extracted from both the tissue and blood samples via a DNeasy Blood and Tissue Kit (QIAGEN), following the manufacturer's recommendations with minor alterations (see supplementary information, Appendix A, Supplementary material online) for more information on DNA extraction, at dedicated molecular ecology laboratories at the National University of Singapore (for *C. galerita s.l.*) and The University of Hong Kong (for outgroups). Once total genomic DNA had been extracted, its quality was assessed by either nanodrop or qubit fluorescence to ensure extracts met quality and quantity thresholds for sequencing. Finally, all samples were sent to BGI (Hong Kong) for 150 bp paired-end PCR-free library preparation, with an average insert size of 350 bp, constructed using the MGIEasy PCR-Free DNA Library Prep Set and sequenced to 10–30× coverage (∼11–33Gb) on the DNBSEQ™-G400 platform (supplementary table S2, Appendix B, Supplementary Material online).

### Sequence Processing

For processing and downstream analyses, the IT Center for Science (CSC), Finland, provided access to the cluster computer Puhti. Here, sequencing reads were trimmed of adaptors using AdapterRemoval 2.3.3 (Schubert et al. 2016) and mapped to the reference genome of the Palm Cockatoo, *Probosciger aterrimus* (Gmelin 1788) (GenBank accession no. GCA_013397665.1; Feng et al. 2020), using BWA mem 0.7.17 (Li and Durbin 2010). The program SAMtools 1.16 (Li et al. 2009; Danecek et al. 2021) was used to combine, sort, and index bam files containing paired-end and merged reads of each specimen independently, before a combination of Picard 2.27.5 (broadinstitute.github.io/picard) and SAMtools 1.16 was used to add read groups, mark duplicates, and sort and index the combined bam file of each specimen respectively. The GATK 4.3 (McKenna et al. 2010) best practices pipeline was used to process the bam files of all specimens and call variants (see supplementary information, Appendix A, Supplementary material online) for a summary of the pipeline applied. Variants were then mapped to a pseudochromosome layout of the *P. aterrimus* genome (see supplementary information, Appendix A, Supplementary material online) for further details on reference genome selection and layout construction using Chromosomer (Tamazian et al. 2016), as implemented through Python programming language 2.7.2 (www.python.org), and the combined variant call format (VCF) file was sorted using VCFtools 0.1.16 (Danecek et al. 2011). BCFtools 1.16 (Danecek et al. 2021) was then used to generate VCF files of each chromosome independently. Thereafter, VCFtools 0.1.16 was again used to apply basic filtering to each chromosome, selecting only SNPs, to limit missing data (−max-missing 1) and setting minimum (−min-meanDP 5) and maximum read (−max-meanDP 35) depth filters, before generating Plink 1.90 (Chang et al. 2015) compatible inputs. Finally, Plink 1.90 was used to merge autosomes, select specific individuals, carry out subsequent minor allele frequency (MAF; to ensure minor alleles are at least present in ≥ 2 individuals) and linkage disequilibrium (LD; window size = 50 kb, step size [variant count] = 5, *r*^2^ threshold = 0.2) filtering and, where required, designate a targeted amount of random SNPs for analyses to generate compatible and relevant output files. All relevant VCF files and analytical input files can be found on the Dryad Digital Repository (doi.org/10.5061/dryad.ghx3ffbxc; Sands et al. 2024), while computational scripts are available on GitHub (github.com/AFSands).

### Phylogenomic Inference

To determine the relationships between individuals and assess the monophyly of *C. galerita* within *Cacatua* or to identify any cryptic interspecific diversity to be excluded from downstream intraspecific analyses, IQ-TREE 2.2.0.3 (Minh et al. 2020) was used to generate a maximum likelihood (ML) phylogeny. To construct the relevant input, a VCF file of 2,221,243 SNPs and 99 specimens from Plink 1.90 (excluding the *C. moluccensis* singleton to improve the reliability of calls through MAF) was converted to fasta format using vcf2phylip 2.8 (Ortiz 2019; github.com/edgardomortiz/vcf2phylip) as implemented in Python 3.10.6 (www.python.org). Thereafter, IQ-TREE 2.2.0.3 was run for 10,000 bootstrap replicates, selecting the -bnni flag to reduce the overestimation of branch support and applying the GTR + ASC best-fit model of sequence evolution, as recommended by IQ-TREE 2.2.0.3 for SNP datasets (www.iqtree.org/doc/Substitution-Models). The consensus phylogeny with bootstrap support (BS) values was then viewed through FigTree 1.4.4 (Rambaut 2018) and rooted to *C. alba* based on White et al. (2011), Selvatti et al. (2022), and Smith et al. (2023). Additionally, to affirm the phylogenetic patterns observed, the same –bfile Plink 1.90 outputs of 99 specimens and 2,221,243 SNPs used to generate the VCF for the ML phylogeny were employed to perform an interspecific principal component analysis (PCA) for the first three components using Plink 2.0 (Chang et al. 2015).

### Intraspecific Structure and Admixture

To determine population structure, visualise shared genetic compositions, and assist with the establishment of ESUs in *C. galerita*, multiple analyses were run on intraspecific diversity (*n* = 88; excluding *C. galerita* from south-eastern New Guinea and outgroups). Phylogenomic and taxonomic patterns of relationships in intraspecific diversity were estimated from the ML phylogeny and supplemented with the results of ordination analyses (supplementary fig. S1, Appendix C, Supplementary Material online).

Herein, first, an intraspecific PCA of 1,745,807 SNPs, from –bfile Plink 1.9 outputs, was performed for the first three components using Plink 2.0 to assess the genetic similarity among specimens and identify population groupings. Second, to determine potential introgression or admixture between genetic clusters (*K*) or plausible ESUs of *C. galerita,* admixture plots were generated with ADMIXTURE 1.3 (Alexander et al. 2009) to display shared genetic composition between specimens using the same Plink 1.90 outputs as for the PCA. To test for congruence among assumptions, 10 replicates of plots, applying random seeds, under the assumption of between 1–6 *K* were constructed (where *K* = 6 equated to a maximum reasonable number of historically recognised subspecies possibly sampled). To validate the most likely *K* assumption, cross-validation (c.v.) errors for each plot were calculated and averaged between the ten replicates for each respective *K* assumption (i.e. *K* = 1–6). Here, lower c.v. errors are seen as support of a more credible *K*. Finally, to relate the two analyses and provide provisional ESU assessments, the intraspecific PCAs of *C. galerita* were recoloured according to the most credible *K* assumptions for comparison.

### Demographic History

To gain a better perspective on historical demographic changes relevant for determining evolutionary settings, which may be preferential to different *C. galerita* ESUs and *Cacatua* species, we created pairwise sequentially Markovian coalescent (PSMC; Li and Durbin 2011) plots for all individuals with ≥ 18× genomic coverage (Nadachowska-Brzyska et al. 2016; Irestedt et al. 2019) to observe the effective population size (*N*_e_) over time. Here, BQSR recalibrated bam files (supplementary information, Appendix A, Supplementary Material online), sorted, and indexed with SAMtools 1.16 (see Methods: Sequence processing), were used to create consensus sequences in FASTQ format for each individual using the SAMtools 1.16 “mpileup” and BCFtools 1.16 “call” commands (Danecek et al. 2021) and vcfutils.pl (“vcf2fq” option). Filters of –C 50, –q 30, and –Q 20 were applied and only sites where the read depth was between double and no less than one-third of the average read depth were included. The parameters for the PSMC analyses were set to –N30 –t5 –r5 –p 4 + 30 × 2 + 4 + 6 + 19 (following those used on other Psittaciformes; Irestedt et al. 2019). Moreover, a generation time of 13.8 year (BirdLife International 2023) and a generational mutation rate of 1.05 × 10^−8^ (from an average of two Psittaciformes with similar generation times to *C. galerita*; Martini et al. 2021; Bergeron et al. 2023) were applied. We repeated our PSMC analyses for 100 bootstrap replicates to obtain an understanding of the measure of uncertainty around parameter estimates.

### Phylogenetic Dating, ESU Delimitation and Biogeographic Contextualization

To place intraspecific evolutionary relationships and divergence events in *C. galerita* into a temporal context, a dated phylogeny was constructed with the package SNAPP 1.6.1 (Bryant et al. 2012) in BEAST 2.7.5 (Bouckaert et al. 2019) following PSMC-derived divergence dates (as also validated using SMC++; Terhorst et al. 2017; see supplementary information, Appendix A, Supplementary Material online). Divergences in historical *N*_e_ signatures (i.e. demographic history) are proposed to correlate to evolutionary divergence events among lineages (Mather et al. 2020) and have been widely used in framing the evolutionary histories thereof (Marchi et al. 2021; also see e.g. Song et al. 2021; Andersson et al. 2024). Moreover, this approach allowed us to incorporate a robust mutation-rate-derived calibration to a SNP-based phylogeny given the sparse and poor fossil record for cockatoos (see supplementary information, Appendix A, Supplementary Material online). Here, a VCF subset of 10,000 randomly selected SNPs from Plink 1.90, specifically derived from 34 selected *C. galerita* ingroup specimens and two *C. alba* outgroup samples, was used to create an XTML input file following Matschiner (2018) and the Ruby script provided therein (github.com/mmatschiner/tutorials/blob/master/divergence_time_estimation_with_snp_data). A log-normal distributed prior for the divergence of *C. galerita* from Western Australia and the Tiwi Islands (Northern Territory) from *C. galerita* from Tasmania, South Australia and New South Wales (MRCA = 232 thousand years ago “Kya”, 95% highest posterior density “HPD” = 466–90 Kya) was used to date the initial divergence in the species (see supplementary information, Appendix A, Supplementary material online) for added information on calibration and dating methods. For congruence, 40 independent MCMC simulations were run for between 518,000–548,000 generations, sampling every 500 generations. LogCombiner (BEAST 2.7.5 package) was used to combine log and tree files (applying a 10% burn-in on each simulation) and validation of convergence and mixing of the combined files were assessed in Tracer 1.7.1 (Rambaut et al. 2018) to ensure that all effective sample size values were >200. Trees were subsequently summarised in TreeAnnotator (BEAST 2.7.5 package) with no additional burn-in.

Phylogenetically supported subspecies or ESUs were then identified by subjecting the dated phylogeny to the Generalised Mixed Yule Coalescent (GMYC; Pons et al. 2006) and Bayesian implementation of the Poisson tree processes (bPTP; Zhang et al. 2013) delimitation methods online using default settings (species.h-its.org). The bPTP method was run with and without outgroup taxa included and the GMYC method was run under single and multiple thresholds. Additionally, to further explore the level of divergence between delimited ESUs, 1D site frequency spectrum (SFS) files for each regional/subregional group of *C. galerita* were generated through ANGSD 0.940 (Korneliussen et al. 2014). Herein, the BQSR recalibrated bam files (from the GATK 4.3 pipeline; see supplementary information, Appendix A, Supplementary Material online) of all representative *C. galerita*, with the *P. aterrimus* reference genome used as the ancestral reference and a contig map and position file to only include autosomes, were used following Momigliano et al. (2021; see scripts for ANGSD settings). The SFS outputs were run through R 4.2.2 (R Core Team 2022) using a script developed by Momigliano et al. (2021; github.com/Nopaoli/Demographic-Modeling/tree/master/Diversity_fromSFS) to calculate nucleotide diversity (*π*) as well as several other indices, including Tajima's *D* (supplementary table S3, Appendix B, Supplementary Material online), for each regional/subregional group of *C. galerita*, before being used to compute pairwise SFS between regions/subregions using winsfs (Rasmussen et al. 2022). The 2D SFS outputs were run through a further Momigliano et al. (2021) R script to calculate absolute sequence divergence (*d_xy_*), available through the same link. Thereafter, genetic distances, calculated as the pairwise net nucleotide differences (*d_a_*), were also determined for each regional/subregional grouping, using the formula of Nei and Li (1979): *d_a_* = (*d_xy_* − (*π_x_* + *π_y_*)) ÷ 2. To place divergence levels into further context, additional comparisons were made between the regional/subregional groupings of *C. galerita* and other *Cacatua* species (i.e. outgroups based on the ML phylogeny) following the same methods.

To help contextualise the biogeographic and evolutionary history of *C. galerita*, we supplemented the above with modeling estimated effective migration surfaces (EEMS; Petkova et al. 2016). The EEMS analysis plots areas of heightened and lowered gene flow across the landscape, bringing perspectives on historical and contemporary breaks to gene flow. Here, 10,000 randomly chosen SNPs from the set of 1,745,807 used to create the intraspecific PCA (see intraspecific structure and admixture) were used to estimate the migration surface across the species' distribution range in Australia and southern New Guinea with 20 million MCMC iterations and 10 million burn-in iterations. Two independent EEMS analyses were run with different random seeds to ensure convergence of the MCMC chains. The migration surfaces were plotted with the R-package runEEMS.plot (github.com/dipetkov/eems) using R 4.2.2. The EEMS analyses incorporate and assume population structure is consistent with isolation-by-distance (IBD). Thus, to further assess the levels of genetic diversity that can be attributed to geographic distance we tested for IBD using the distance-based redundancy analysis (db-RDA; Legendre and Anderson 1999; Legendre and Fortin 2010), similar to Sands et al. (2019), with the package vegan 2.6-4 (Oksanen et al. 2022) in R 4.2.2. Given our phylogenomic signals, we tested for IBD among *C. galerita* excluding *C. g. triton* from south-eastern New Guinea (i.e. Oro Province). Pairwise genetic and geographic distance (km) matrices were calculated with the set of 1,745,807 SNPs mentioned above and GPS coordinates of specimens using ngsDist (Vieira et al. 2016) and Geographic Distance Matrix Generator 1.2.3 (Ersts 2006).

In addition to EEMS and IBD, and to further contextualise the biogeographic history of *C. galerita*, ancestral areas were estimated using BioGeoBEARS 0.2.1 (Matzke 2013a) through RASP 4.3 (Yu et al. 2020). Here, areas were estimated under six different biogeographical models (i.e. DEC, DEC + J, DIVA-like, DIVA-like + J, BayArea-like, and BayArea-like + J) across the ML and dated SNAPP phylogenies (see Matzke 2013b, 2014). The corrected Akaike information criterion (AICc; Sugiura 1978; Hurvich and Tsai 1989) was used to select the best-fit model from analyses with 13 predefined areas (i.e. all regional/subregional designations noted in Fig. 1 plus “Wallacea” for *C. alba* and *C. sulphurea* outgroups) and maximum areas set at three. Thereafter, the results from the best supported model were plotted onto the phylogenies. The ML phylogeny results were additionally transferred to corresponding nodes on the dated SNAPP phylogeny for comparative purposes.

### Bioacoustic Analyses

**Fig. 2.**
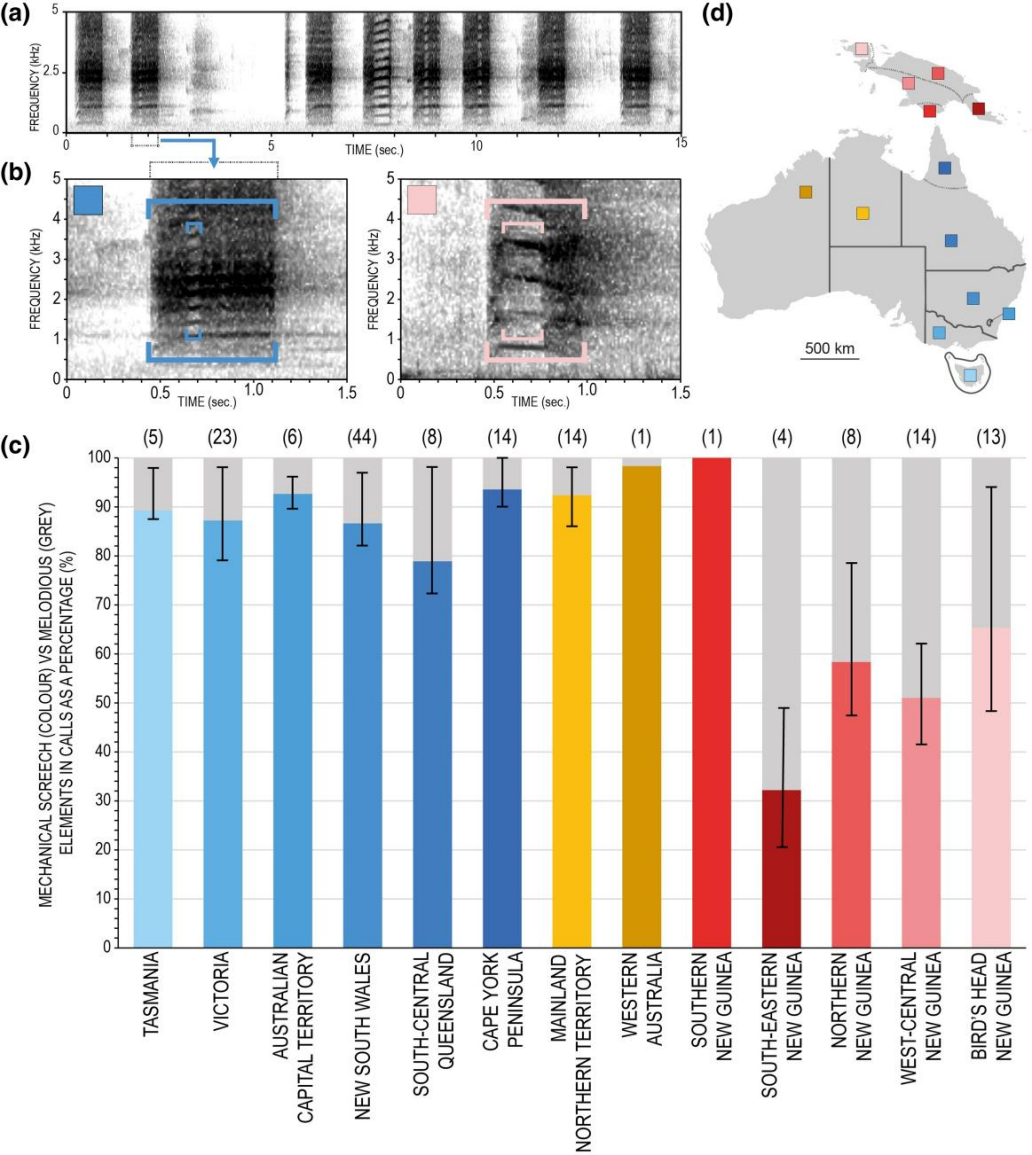
Bioacoustic analyses and results. a) A typical recording of eight calls of *C. galerita* from New South Wales, Australia. b) An enlarged call from the above alongside a call from the Bird's Head subregion, New Guinea indicating bioacoustic measurements: Larger square brackets indicate call duration, while smaller square brackets cover clear harmonics (> ∼0.3 kHz) representing melodious elements, contrasting with blurry and dense mechanical-sounding sections. c) A bar graph shows the percentage of mechanical-sounding vs. melodious elements in calls analysed from 13 regions/subregions across New Guinea and Australia (also see supplementary table S4, Appendix B, Supplementary Material online). Whiskers in bars depict the 75% and 25% quartiles among analysed calls. Numbers in brackets above bars reflect the number of recordings analysed in each region/subregion. The bars are coloured according to d) the map key.

To provide additional support for intraspecific and/or cryptic interspecific diversity patterns in *C. galerita*, as well as greater resolution/data points for distribution range assessments thereafter (where patterns can be linked with genomic lineages), we analysed 135 recordings of *C. galerita* downloaded from xeno-canto (www.xeno-canto.org) and 300 from eBird (www.ebird.org) representing the natural distribution range. First, we filtered out recordings duplicated between the databases. Second, we removed recordings with poorly defined geographical origins, conflicting background noise (from other birds or Psittaciformes with similar call types) and those with poor spectrogram resolution or less than three calls. Finally, we additionally avoided recordings taken by the same recorder at the same location on the same day to reduce the likelihood of pseudoreplication. As *Cacatua* produce a variety of calls and have the ability to learn or mimic calls, comparing call types remains challenging. However, calls are made up of both “mechanical-sounding” and “melodious” elements useful for comparisons. We defined melodious elements as reflected by portions with clear harmonics spaced at > ∼0.3 kHz intervals in spectrograms, while the mechanical-sounding screeching elements were designated as densely blurred grey portions (Fig. 2a and b). Here, measurements of duration of melodious elements in calls (as a percentage ratio of total call time) were made between groups. We used Raven Lite 2.0.5 (K Lisa Yang Center for Conservation Bioacoustics 2024) to measure the duration of ten clearly defined calls from each recording (or as close to ten as possible if the recording had less than ten clear calls) and the duration of the two elements therein. In total 155 recordings were used in the final dataset (Fig. 1; supplementary table S4, Appendix B, Supplementary Material online). We pooled measurements from recordings from areas across Australia and New Guinea to work out regional/subregional averages, which were plotted for comparison, along with the 25% and 75% quartiles, respectively. As added support for linking or excluding genomic lineages to/from the observed bioacoustics patterns of regions/subregions lacking genomic sampling in New Guinea, a Kruskal–Wallis test (KWt) with Bonferroni correction, followed by a post hoc Dunn's test, was completed online (www.statskingdom.com) after assessing for normality (Shapiro–Wilk test of normality; *W* = 0.8222, *df* = 148, *P* < 0.001). We compared the signatures in these regions/subregions in New Guinea to themselves and with pooled regions/subregions representing key lineages from our phylogenomic analyses.

## Results

### Phylogenomic Inference

**Fig. 3.**
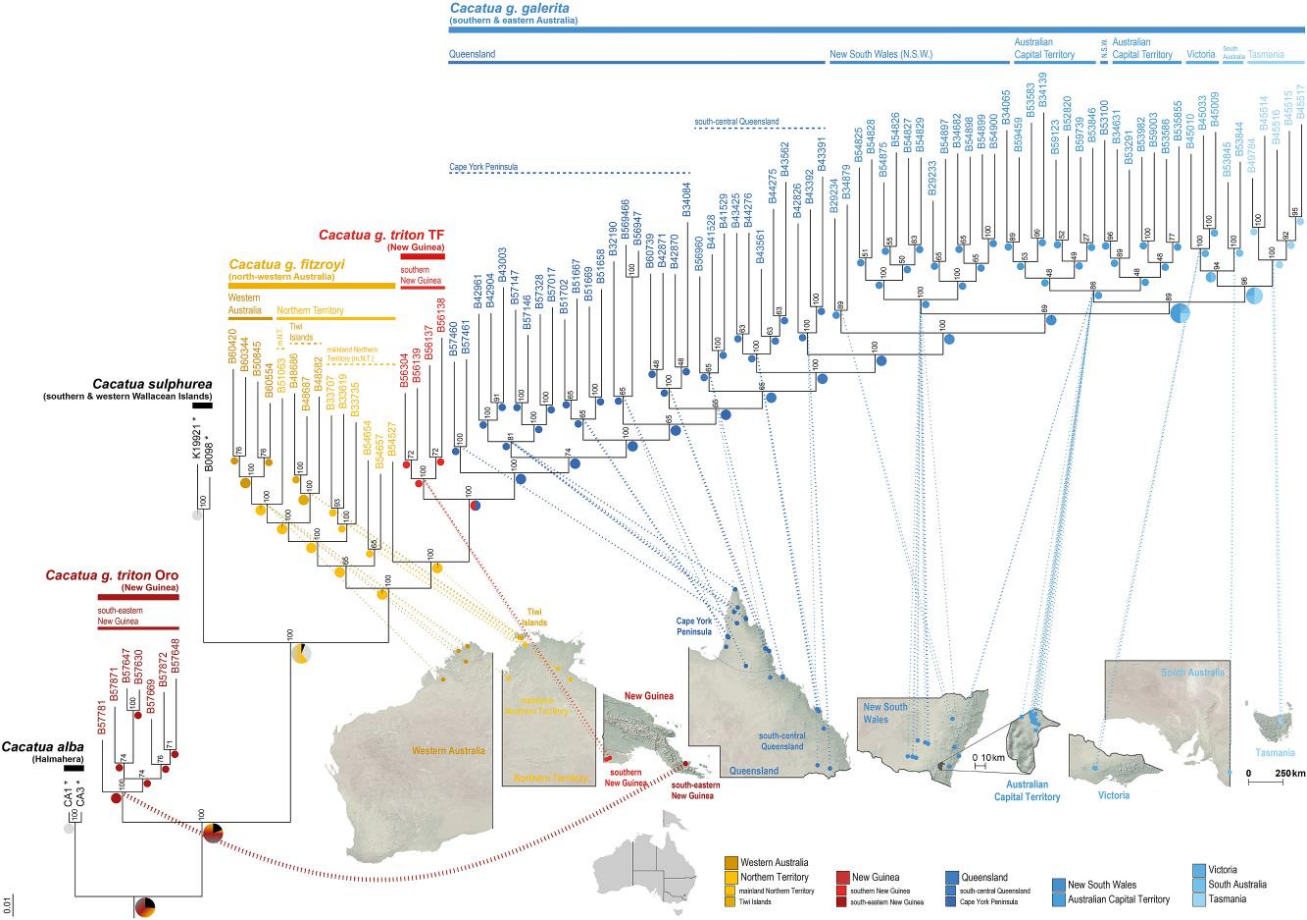
SNP-based, 10,000 bootstrap replicate, ML phylogeny generated with IQ-TREE 2.2.0.3 for 95 *C. galerita*, two *C. sulphurea* and rooted with two *C. alba* specimens (*n* = 99). Bootstrap support values are shown at each node. Voucher numbers of all specimens are shown at tips (also see supplementary table S2, Appendix B, Supplementary Material online), where those marked with asterisks (*) denote feral or captive origin. Clade provenance of individuals is linked to regional/subregional maps of Australia and New Guinea via dotted lines. Pie charts at nodes show estimated ancestral areas per the DEC + J model as coloured according to the key, barring “Wallacea” (dark grey) for outgroups and other/unresolved ancestral ranges (black). Ancestral area estimates with portions containing ≥ 2 region/subregion combinations are gradient-filled according to the colour codes. Abbreviations: TF = Trans-Fly ecodomain of southern New Guinea and Oro = Oro Province of Papua New Guinea. Taxonomy is that applied at the study's commencement.

The ML phylogeny generated with IQ-TREE 2.2.0.3 reveals paraphyly in some *C. galerita* subspecies and in *C. galerita* as a whole (Fig. 3). First, specimens nominally of *C. g. triton* form two deeply divergent clades. One clade includes individuals from southern New Guinea in the Trans-Fly ecodomain (hereafter *C. g. triton* TF) and is sister to a clade of eastern Australian *C. g. galerita*. The other clade comprises individuals from Oro Province along the North coast of south-eastern New Guinea (hereafter *C. g. triton* Oro) and is nestled among the outgroup taxa (Fig. 3). Second, specimen B54527 (ANWC), nominally the south-easternmost sample of *C. g. fitzroyi* in the study and so geographically closest to *C. g. galerita*, is sister to *C. g. galerita* and *C. g. triton* TF, so rendering *C. g. fitzroyi* paraphyletic (Fig. 3). These relationships are supported, as are most nodes in the phylogeny (83 of the 98 nodes being validated by BS ≥ 65; Fig. 3).

Excluding *C. g. triton* Oro, the intraspecific diversity in the phylogeny shows the subclades comprising *C. g. galerita* and *C. g. fitzroyi* (except B54527) as depicting structure corresponding to clinal patterns of latitudinal (*C. g. galerita*) or longitudinal (*C. g. fitzroyi*) gradients (Fig. 3). The db-RDA of this group found significant IBD within *C. galerita* excluding *C. g. triton* Oro (adj. *R*^2^ = 0.077, *P* < 0.001; supplementary information, Appendix A, Supplementary Material online).

### Intraspecific Structure and Admixture

**Fig. 4.**
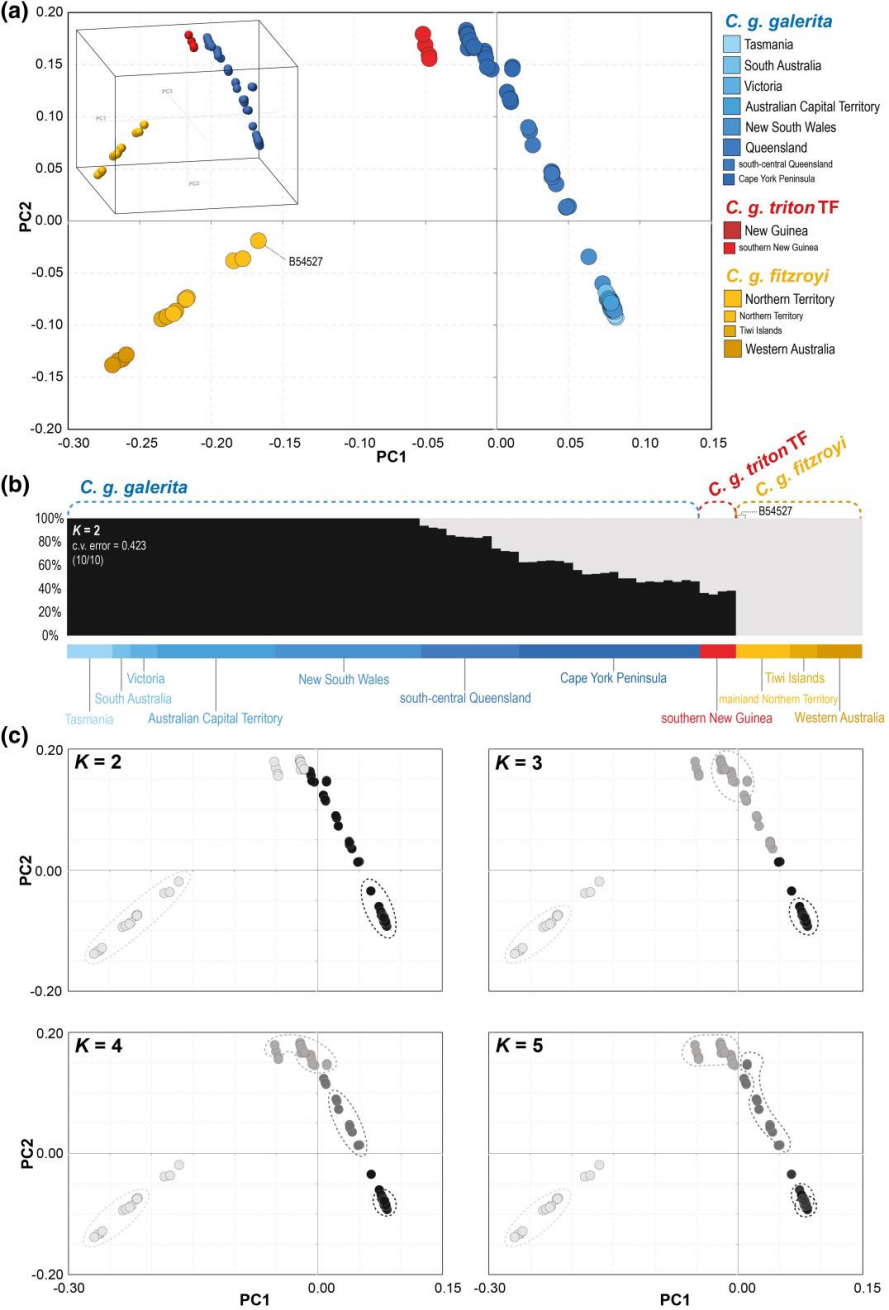
a) Intraspecific PCA of 88 *C. galerita* specimens from 1,745,807 SNPs generated with Plink 2 and coloured according to the key (TF abbreviation as in Fig. 3). The inset shows the first three components in 3D space. b) Most favoured admixture plot (genetic clusters; *K* = 2) among values of *K* from 1–6 calculated with ADMIXURE 1.3 using the same SNP dataset. The c.v. error and the frequency of the overall admixture plot observed across 10 replicates are noted in the upper left portion of the plot. c) Greyscale recoloured replicate PCAs by the most frequent admixture plot pattern for *K* = 2–5 (supplementary fig. S2, Appendix C, Supplementary Material online). Each dot represents a specimen coloured by the cluster of highest affinity in each scenario. Dashed lines encircle individuals with ≥ 95% affiliation to a specific *K*.

Interspecific PCA clusters (supplementary fig. S1, Appendix C, Supplementary Material online) supports the relationships identified in the ML phylogenomic analysis (Fig. 3). Therefore, we treat *C. g. triton* Oro as interspecific diversity and only *C. g. triton* TF is incorporated in subsequent intraspecific analyses within *C. galerita*. The intraspecific PCA of *C. galerita* depicts two broad clusters; one containing *C. g. galerita* and *C. g. triton* TF and the other containing *C. g. fitzroyi* (Fig. 4a). Within each cluster similar longitudinal and latitudinal gradients are observed as in the phylogenomic analysis (Fig. 3).

Further assessments of population structure through admixture plots (supplementary fig. S2, Appendix C, Supplementary Material online) under *K* = 1–6 suggest *K* = 2 as the most reliable (Fig. 4b; supplementary table S5, Appendix B, Supplementary Material online), while reductions in reliability and/or reproducibility can be observed among the rest (supplementary fig. S2, Appendix C and supplementary table S5, Appendix B, Supplementary Material online). Under *K* = 2, all specimens nominally of *C. g. fitzroyi* from northern and north-western Australia form a unique cluster (Fig. 4b). Similarly, *C. g. galerita* specimens from south-eastern Australia (i.e. Tasmania, South Australia, Victoria, the Australian Capital Territory, and New South Wales) form a distinct cluster, having no genetic overlap with *C. g. fitzroyi* (Fig. 4b). However, more northern regions/subregions of *C. g. galerita* from south-central Queensland and the Cape York Peninsula and *C. g. triton* TF from southern New Guinea show admixture at varying levels with both of the two primary clusters (Fig. 4b). When shading the most likely *K* affiliations of individuals and encircling those sharing ≥ 95% genetic identity to a specific *K* (under *K* = 2–5) on the intraspecific PCA (Fig. 4c), *C. g. triton* TF almost exclusively groups with individuals from the Cape York Peninsula, Queensland.

### Demographic History

**Fig. 5.**
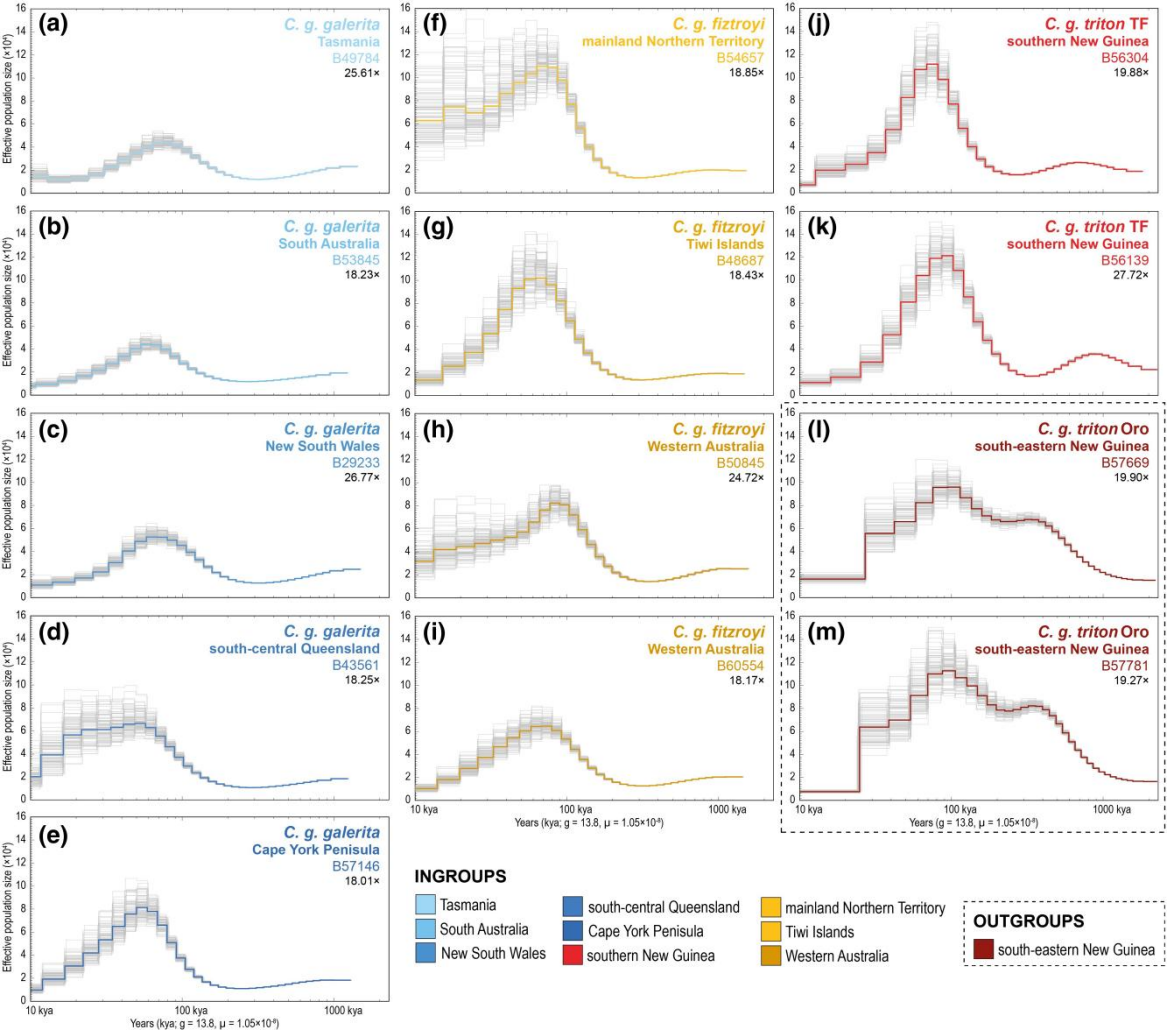
PSMC plots for individuals sequenced with ≥ 18× (±19.8 Gb) genomic coverage, showing effective population size (*N*_e_) over time from 2.5 million years ago (Mya) to 10 thousand years ago (Kya). Abbreviations TF and Oro are as in Fig. 3. Thin grey lines represent the 100 bootstrap replicates in each plot, while colour lines denote the consensus among replicates. Regional/subregional localities, Australian National Wildlife Collection (ANWC) voucher numbers and genomic coverage are also noted in each plot. Colour coding is as in other figures and reflected in the key. Taxonomy is that at the commencement of the study.

Among the PSMCs of specimens attributed to the ingroup clade of *C. galerita* (i.e. all except *C. g. triton* Oro), PSMCs reflect populations undergoing heightened expansion in *N*_e_ beginning just over 200–300 Kya to ∼50–100 Kya (Fig. 5a–k). This expansion event is followed by a steady contraction event beginning ∼40–90 Kya that lasted at least until the end of the Pleistocene (Fig. 5a–k). In comparison, PSMCs from specimens representing *C. g. triton* Oro (i.e. those nestled among the outgroups; Fig. 3) show patterns associated with populations depicting an earlier, large expansion in *N*_e_ beginning just over 1 million years ago (Mya) and persisting to ∼350–300 Kya (Fig. 5l and m). This expansion event is then followed by a short contraction event and then another large expansion event ∼210 Kya, which corresponds with the initial expansion seen in the main clade containing the ingroup *C. galerita*, as mentioned first. At ∼90–85 Kya *C. g. triton* Oro also began to contract. However, around ∼28–20 Kya, a sudden, and sharp fall in *C. g. triton* Oro *N*_e_ can be observed which is not present among the PSMCs of the ingroup *C. galerita* from the main clade (Fig. 5l and m vs. 5a–k).

Within *C. galerita* (excluding *C. g. triton* Oro), PSMCs generated from specimens from southern New Guinea (i.e. *C. g. triton* TF), Western Australia and all subregions across the Northern Territory and Queensland, populations reflect greater expansions in *N*_e_ beginning ∼310–210 Kya and thus typically more pronounced declines in comparison to those from more southerly regions of the range between ∼87–49 Kya and 10 Kya (Fig. 5a–d vs. 5e–k). Moreover, with the exception of specimen B49874 from Tasmania (Fig. 5a), there is little to no indication of population expansions in the most recent time bracket at 10–20 Kya (Fig. 5) and all effective population sizes remain generally low compared to the Middle and Late Pleistocene. However, caution should be taken when evaluating trends in this most recent time bracket due to the unreliability of PSMCs in spanning the last 1,000 generations. These PSMC results are matched by Tajima's *D* values among populations of all regions/subregions, which are either close to zero or positive. These *D* values reflect stable populations or those expected to have gone through sudden population contractions (supplementary table S3, Appendix B, Supplementary Material online).

### Phylogenetic Dating, ESU Delimitation and Biogeographic Contextualisation

**Fig. 6.**
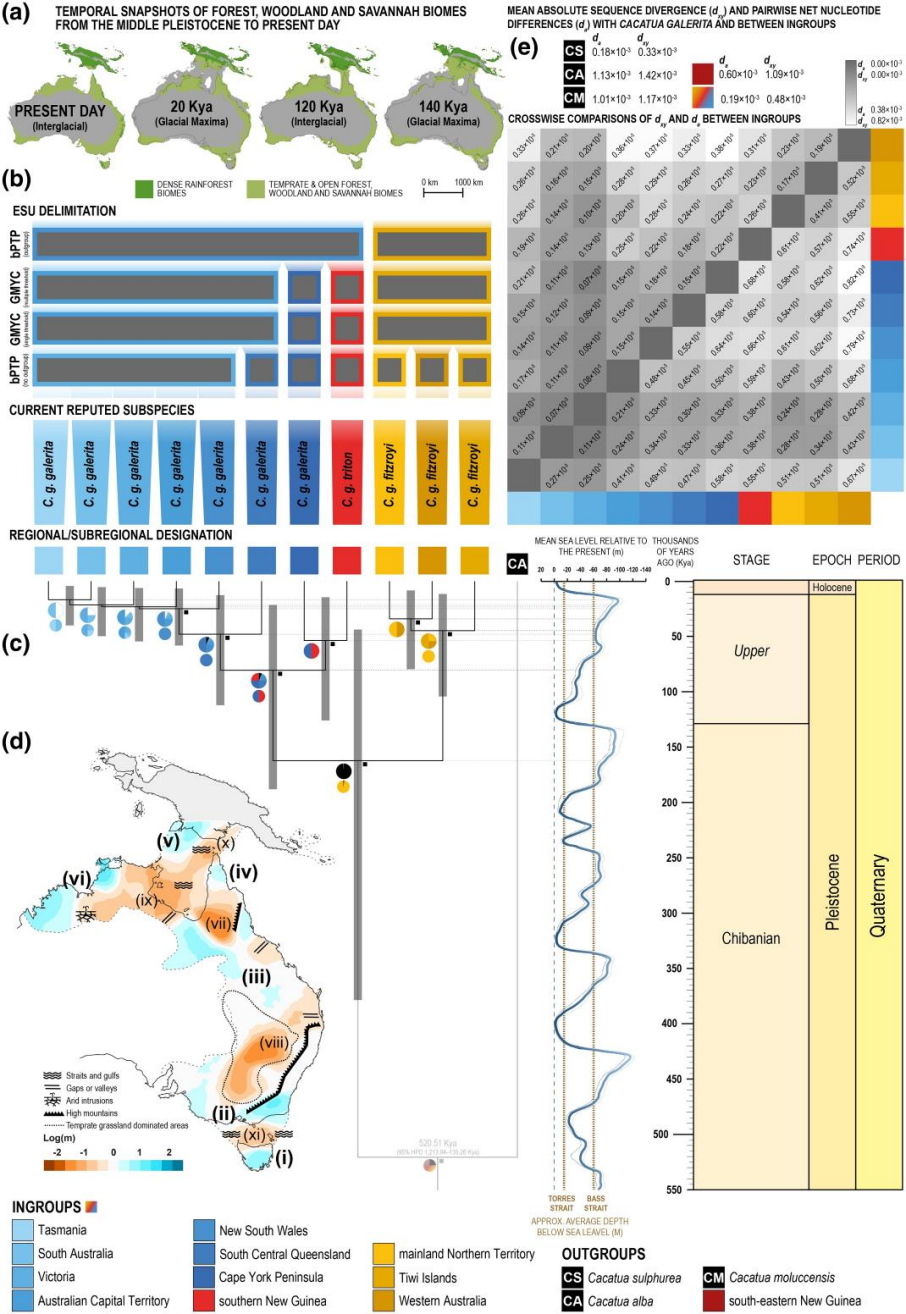
a) Dense rainforest vs. temperate and open forest, woodland, and savannah biomes from the Middle Pleistocene to present-day (adapted from Allen et al. 2020). b) Evolutionarily significant unit (ESU) delimitation results vs. currently recognised subspecies. c) The dated SNAPP phylogeny (see Materials and Methods) of the *C. galerita* complex. CA notes the outgroup *C. alba*. Small black squares at nodes reflect significant posterior probabilities (PP) of relationships. Grey bars show 95% confidence intervals of divergence dates. Dates are aligned to Quaternary sea levels (Berends et al. 2021) and a geological time scale at the right. Pie charts at nodes display ancestral areas estimated by DEC + J model (Large pies = modelled from the dated phylogeny; small pies = from Fig. 3 for comparison). The colouring of pie charts is the same as noted in Fig. 3. d) Estimated effective migration surfaces (EEMS) with commonly hypothesised biogeographic boundaries superimposed (see Materials and Methods). Areas of heightened (blue) or neutral (white) gene flow (i–vi; bold) and lowered (orange) gene flow (xii–xi) are shown. Darker shading of colours (blue/orange) indicates more extreme cases of each scenario (see subkey). The dashed line encloses the native distribution range of *C. galerita* as sampled (Fig. 1). Greyed-out areas reflect parts of New Guinea and Aru Islands not assessed by EEMS. e) Averaged absolute sequence divergence (*d_xy_*) and pairwise net nucleotide differences (*d_a_*) between specimens of *C. galerita* (excluding *C. g. triton* Oro) as a whole and compared to outgroup taxa (above), with a matrix (below) of individual comparisons between regions/subregions shaded according to *d_xy_* and *d_a_*, below and above the diagonal, respectively. The overall figure colour scheme follows that used in other figures and as also guided by the key.

The intraspecific structure within the dated SNAPP phylogeny, as generated through BEAST 2.7.5 using PSMC-derived divergence dates, is well supported with only three nodes' posterior probabilities (PP) < 0.95 (Fig. 6c). These nodes correspond to the divergences among *C. galerita* populations within south-eastern Australia from Tasmania, South Australia, Victoria, and the Australian Capital Territory. These populations geographically neighbor each other and show higher levels of genetic similarity (Figs. 4 and 6; supplementary fig. S2, Appendix C, Supplementary Material online). Additionally, some discordances with the ML phylogeny are observed. First, within *C. g. fitzroyi*, samples from the Tiwi Islands are sister to other samples from the mainland Northern Territory and Western Australia. Second, within *C. g. galerita*, samples from the Cape York Peninsula are sister to *C. g. triton* TF rather than to *C. g. galerita* from more southerly Australian regions (Figs. 3 and 6c). These discordances are likely due to subsampling of individuals for the dated phylogeny and/or possibly the reduced number of SNPs used (supplementary table S2, Appendix B, Supplementary Material online). The dated phylogeny shows two broad clades. One clade includes specimens of *C. g. fitzroyi* from Western Australia and the Northern Territory (including the Tiwi Islands). The second clade comprises specimens of *C. g. galerita* and *C. g. triton* TF from regions/subregions across eastern Australia (including Tasmania) and southern New Guinea, respectively. The first intraspecific divergence event between these two broad clades likely occurred 162.03 Kya (95% HPD = 378.46–43.52 Kya; Fig. 6c), followed by the divergence between the combined cluster consisting of *C. g. galerita* from the Cape York Peninsula and *C. g. triton* TF on the one hand, and all other eastern Australian *C. g. galerita* on the other hand, at ∼53.36 Kya (95% HPD = 125.62–14.32 Kya; Fig. 6c). Thereafter, divergence events occur at regular intervals over the latter stages of the Quaternary period (i.e. Late Pleistocene and Holocene; Fig. 6c).

The ancestral area estimations, calculated through BioGeoBears 0.2.1 (as implemented in RASP 4.3), found the DEC + J model, which allows for founder events, to fit the ML and dated SNAPP topologies best. The common ancestor of *C. galerita* is found to have had a distribution that may have corresponded with the mainland Northern Territory of Australia (Figs. 3 and 6c). This ancestral distribution is shared with the common ancestor of the first broad clade incorporating *C. g. fitzroyi* specimens from Western Australia, Tiwi Islands, and the mainland Northern Territory (Fig. 3), though estimations on the dated phylogeny (which exclude the independent ANWC B54527 lineage that is shown to be possibly derived from admixture; supplementary fig. S2, Appendix C, Supplementary Material online) indicate that the ancestor of this clade could have had a broader ancestral distribution across more of northern and north-western Australia (Fig. 6c). The common ancestor of the second broad clade, comprising all *C. g. galerita* specimens and *C. g. triton* TF, evolved from a common ancestor shown to share a distribution inclusive of north-eastern Australia (i.e. Queensland and possibly New South Wales) and southern New Guinea across the Torres Strait (Fig. 6c). Thereafter, divergences in this clade reflect a southward range expansion with Tasmania being the most recently colonised (Fig. 6c).

Phylogenetic delimitation methods of ESUs show varying results. The bPTP method, with the outgroup omitted, delivered seven ESUs with limited concordance with past or currently accepted subspecies. Most conservatively, the bPTP method, including the outgroup, returned only two ESUs, which broadly correspond to the two Australian subspecies currently accepted (i.e. *C. g. galerita* and *C. g. fitzroyi*; Fig. 6b; supplementary fig. S3, Appendix C, Supplementary Material online). Along a spectrum between these two results, the GMYC yielded four ESUs irrespective of the threshold used (i.e. single vs. multiple; Fig. 6b; supplementary fig. S3, Appendix C, Supplementary Material online). These four ESUs are consistent with the current subspecies (i.e. *C. g. galerita*, *C. g. triton* TF and *C. g. fitzroyi*), except that it supports recognition of an ESU from the Cape York Peninsula which may reflect the currently synonymised *Cacatua galerita queenslandica* (Mathews 1912) (supplementary table S1, Appendix B, Supplementary Material online), as recognised by Forshaw and Cooper (2002).

The *d_xy_* and *d_a_* (Fig. 6e) do not correspond well with less conservative phylogenetic delimitations, but show some congruence with the bPTP (including outgroups) results. Overall both *d_xy_* and *d_a_* depict two broad areas which generally share closer affiliations among regions/subregions; namely one group from mainland Northern Territory, Tiwi Islands and Western Australia and another group from the remainder of the range. However, the results also recognise that there is a gradient of relatedness, with increased similarity between neighboring regions/subregions—especially along the eastern seaboard of Australia into southern New Guinea (Fig. 6e). Additionally, a comparison of *d_xy_* and *d_a_* averages in *C. galerita* with outgroup taxa and *C. g. triton* Oro shows high distinctiveness of *C. g. triton* Oro (Fig. 6e). Interestingly, differentiation between *C. galerita* and introduced *C. sulphurea* of feral origin from Hong Kong is observed to be shallow, with some level of overlap with *C. galerita* intraspecific diversity (Fig. 6e).

The EEMS analysis, which accounts for IBD, shows six areas within the range of *C. galerita* (excluding *C. g. triton* Oro) where gene flow is neutral to heightened (Fig. 6d: Areas i-vi). These six areas are separated from one another by five presumptive barrier zones (Fig. 6d: Areas vii-xi). These barrier zones primarily overlap with areas presently or historically more dominated by non-forest/woodland biomes (e.g. grasslands; DAWE 2012; MPIGA and NFISC 2018; Williams et al. 2021) that are reinforced by hypothesised geological and landscape barriers to gene flow (Fig. 6d; Ford 1987; Eldridge et al. 2011; Bryant and Krosch 2016).

### Bioacoustic Analyses

Comparisons in vocalizations reflect the broader phylogenomic patterns observed. Calls of *C. galerita* across Australia (irrespective of ESU/subspecies designation) contain higher durational proportions of mechanical-sounding than melodious elements in calls, with no major variation among populations (Fig. 2c and d). In New Guinea, vocalizations from the south-eastern subregion, covering the range of *C. g. triton* Oro, contain a higher percentage of melodious portions in calls relative to mechanical-sounding screeching, while the only available recording from the southern subregion, overlapping with *C. g. triton* TF, shows the reciprocal pattern as seen in Australian *C. galerita* (Fig. 2c and d). Recordings from other regions/subregions across New Guinea, not covered in the genomic data, correspond more closely with *C. g. triton* Oro, and with increased duration of melodious elements in calls (Fig. 2c and d). Additionally, our KWt results corroborates these comparisons. No significant differentiation in bioacoustic patterns are found between central-western, northern, and Bird's Head subregions of New Guinea (*H* = 1.277, *df* = 34, *P* > 0.05), while significant differentiation of patterns from these subregions to both *C. g. fitzroyi* from the Northern Territory and Western Australia (*H* = 18.305, *df* = 49, *P* < 0.001) and *C. g. galerita* from across the eastern Australia regions/subregions (excluding Southern Australia due to a lack of bioacoustics data) were found (*H* = 38.338, *df* = 134, *P* < 0.001).

## Discussion

We aimed to assess whether currently recognised subspecies of *C. galerita* represent ESUs, if any cryptic diversity exists and whether previously recognised subspecies require reinstatement in order to provide conservation recommendations. Our genomic and bioacoustic results consistently support the existence of two species, *C. galerita* and *C. triton*, within what has long been considered a single species, *C. galerita.* At the same time, within *C. galerita sensu stricto* (*s.s.*), our analyses identify two subspecies corresponding to ESUs. We now examine these key findings more closely.

### Phylogenomic Relationships and ESUs

First, we detected paraphyly within *C. g. triton*, which is currently the only subspecies of *C. galerita* assigned to New Guinean populations (Beehler and Pratt 2016). This also resulted in paraphyly within *C. galerita* itself as currently construed (Fig. 3). One lineage of *C. g. triton* comprised samples from the Trans-Fly ecodomain of southern New Guinea (i.e. *C. g. triton* TF) and is nested within a larger clade of all other *C. galerita* individuals from Australia. The second lineage, *C. g. triton* Oro, includes samples from the northern coast of south-eastern New Guinea, which in contrast is paraphyletic with respect to other *C. galerita* samples, and is comparatively distantly related to them, especially when the outgroup *C. sulphurea* is included. Further, *C. g. triton* Oro differs in its demographic history, has substantial genetic distance from all other *C. galerita* sampled and recordings from its range convey differing bioacoustic signatures to *C. galerita s.s.* (Figs. 2, 5, and 6e). Our data support recognition of this lineage as a separate species distinct from *C. galerita s.s*.

Accordingly, we propose restoration of the epithet *triton* to species rank as *C. triton* Temminck (1849) for what we have hitherto termed the *C. g. triton* Oro lineage. Following Peters (1937), the type locality of this name, which is currently assigned to all New Guinean populations not just those of the Oro lineage, is Aiduma Island, near Triton Bay, at the neck of the Bird's Head Peninsula in the northern New Guinea subregion. This is much further north-westward than any of our genomic samples (Fig. 1), nonetheless, we make several points here. We note the similarity in bioacoustic signatures between the vocalizations sampled from close to Triton Bay and more distant south-eastern New Guinean localities from where we have genomic data of *C. g. triton* Oro. Moreover, we also note the similarity in vocalizations from Australian regions/subregions with that from southern New Guinea (i.e. that with close proximity to the sampling localities of *C. g. triton* TF) and the significant differentiation in percentage of melody in calls between Australian populations and those in central-western, northern, and Bird's Head sub regions of New Guinea. These observations suggest that *C. triton*, as here recognised, extends over much of the traditionally denser forested parts of New Guinea. The distribution range of *C. triton* narrowly excludes populations from the more savannah-dominated Trans-Fly ecodomain. A caveat to this is that future research involving genomic sampling across New Guinea is encouraged to corroborate this taxonomic treatment.

Second, within the clade comprising *C. galerita s.s.*, our results support two ESUs corresponding to *C. g. fitzroyi* and *C. g. galerita* (the latter here tentatively comprising *C. g. galerita + C. g. triton* TF). These two ESUs are supported by admixture analyses and our PCAs (which depict two broad clusters and favour *K* = 2 respectively; Fig. 4), monophyly (Figs. 3 and 6c), diversity statistics *d_xy_* and *d_a_* (Fig. 6e) and the most conservative ESU delimitation regime (Fig. 6b). A key caveat here is that we have not sampled the Aru Islands populations assigned to *C. g. eleonora*. Given our phylogeographic findings (particularly IBD), our results clearly dictate a need to include the end points of clines in genomic characters in affirming ESUs. Pending sampling of the Aru Islands populations, we provisionally include *C. g. triton* TF within the same ESU as *C. g. galerita* of eastern Australia. Beyond this, we find limited support for any other cryptic diversity across the sampled range warranting reinstatement of long synonymised subspecies or consideration for ESU status (Figs. 3 and 6b and c; also see supplementary information, Supplementary Material online).

### Biogeographic Evolution of *Cacatua galerita*

Our bioacoustic and phylogenomic analyses support *C. galerita s.s.* largely favouring open forest, woodland and savannah environments, although not necessarily exclusively so. In New Guinea, where we find support for two species (albeit with limited genomic sampling), *C. galerita s.s.* seems to be confined to the Trans-Fly ecodomain of southern New Guinea where such biomes persist, while *C. triton* (as here recognised) is in dense rainforest biomes and more narrowly distributed or more temporally short-lived savannah/woodland biomes (Allen et al. 2020).

Our analyses also provide potential biogeographic and demographic perspectives on evolutionary events within *C. galerita s.s.* and the ESUs within it. Quaternary glacial cycling likely played an important part in the evolution and distribution of the whole *C. galerita* complex. While confidence intervals around divergence dates are often broad and means of clock calibration (i.e. mutation rate vs. fossil) should be cautiously interpreted (also see supplementary information, Appendix A, Supplementary Material online), the median dates of divergence achieved should not be ignored and may offer the most reasonable interpretations of the results. The earliest divergence in *C. galerita s.s.*, between *C. g. galerita* and *C. g. fitzroyi*, likely occurred in the Middle Pleistocene at 162.03 Kya (95% HPD = 378.46–43.52 Kya). Ancestral area estimations suggest that the common ancestor of all *C. galerita s.s.* likely occurred in northern Australia and diverged into ancestral *C. g. galerita* and *C. g. fitzroyi* during a glacial maximum. Even considering the broad confidence intervals around the median divergence date, a glacial hypothesis would still reasonably fit well with the ecology of these cockatoos and the phylogenomic signatures observed: The Middle and Late Pleistocene glacial periods are linked with the cooling and drying of climates across the Australasian biogeographic realm. This greatly shifted habitats and exposed land bridges between mainland Australia with Tasmania and New Guinea, respectively (Allen et al. 2020; Williams 2020). Dense forest environments, which dominated parts of the region during warm and wet peaks of interglacials, would have for the most part slowly transitioned into more open forests, woodlands, and savannahs (favoured by *C. galerita s.s.*). These in turn would have been replaced by grasslands in drier parts during glacial maxima, leaving partially fragmented open forest and woodland habitats (Allen et al. 2020). An expansion, followed by reduction and fracture of open forest and woodland habitat, could have caused allo- or parapatric subspeciation in ancestral *C. galerita s.s.*—most likely on either side of the Gulf of Carpentaria. Several authors have proposed the shrinking of forests and woodlands in Australia during this time (Kershaw 1994; Martin 2006; DeSantis et al. 2017; Williams 2020) and similar evolutionary patterns have been documented in other closely distributed bird species favouring open forests and woodlands (e.g. Lee and Edwards 2008; Kearns et al. 2013, 2014; Shipham et al. 2015). If corroborated, the subsequent divergences in regional/subregional subclades of both *C. g. galerita* and *C. g. fitzroyi* between 80.30–16.58 Kya (95% HPD = 187.79–4.18 Kya) could also be viewed to align with the subsequent Late Pleistocene cooler period when lowered sea levels (i.e. exposing land bridges) and habitats would have mimicked those seen roughly 162 Kya (Allen et al. 2020). Oscillations out of interglacial ranges, as open forests and woodlands began to supersede denser forest types during interglacial-glacial transitions, would have allowed for potentially greater range expansions. This might have applied particularly in New Guinea when sea levels were lowered (Berends et al. 2021) and southward along the Australian continent's eastern coast. Thereafter the onset of glacial maxima would have once again restricted populations into refugia prior to rewarming, driving the fine scale clinal population structure observed in the phylogenies and depicted through EEMS (Figs. 3 and 6c and d). As the last glacial maxima receded toward the end of the Pleistocene, open forests and woodlands would have once again expanded, allowing the ESU range expansions' southward (*C. g. galerita*) and east- and westward (*C. g. fitzroyi*; Fig. 6a). These would have permitted refugial populations to reintegrate and it would account for the greater similarity and admixture between neighboring regional/subregional groups. However, the warming climates would have additionally driven the isolation of populations in New Guinea with correlated increased sea levels separating Australia from the rest of Melanesia. Decreases in *N*_e_ across *C. g. galerita* and *C. g. fitzroyi* from around 87–49 Kya to at least 12 Kya (Fig. 5a–k), and even seen in *C. triton*, correspond with the progression of the last glacial period and the overall shrinking of denser rainforest and then open/temperate forest and woodland biomes (Allen et al. 2020; Williams 2020; Adeleye et al. 2021; Rowe et al. 2021). Furthermore, the large expansion in *N*_e_ that began around 210–310 Kya coincides with a period of temporally extended interglacials and less extreme glacial periods ∼250 Kya in comparison to the preceding and succeeding glacial periods (Hughes et al. 2020). In further support of this, it is interesting that our EEMS analysis, which may act as a good starting point for identifying the position and number of local refugia, shows that contemporary terrestrial areas of lowered gene flow often correspond with drier areas either currently dominated by grassland and scrubland biomes or more likely to have transitioned into them during glacial maxima (MPIGA and NFISC 2018; Allen et al. 2020; Williams et al. 2021). Conversely, areas of heightened gene flow correspond with more temporally sustained open and temperate forests, woodlands, and savannah systems (MPIGA and NFISC 2018; Allen et al. 2020; Williams et al. 2021). These patterns and barriers may have been additionally reinforced by aligned geological and landscape elements (e.g. mountains and arid intrusions), which have long been construed to play a role in Australian biogeography (Ford 1987; Eldridge et al. 2011; Bryant and Krosch 2016).

### Implications for Conservation

The support for recognizing two species within what has been treated as *C. galerita,* as well as at least two ESUs within *C. galerita s.s*., has major implications for conservation. First, IUCN assessments and CITES protections need urgent re-evaluation at the species-level. Recognition herein of two species (i.e. *C. galerita,* from Australia and southern New Guinea, and *C. triton* likely from the rest of New Guinea) may warrant appropriate adjustment of priorities of protection/conservation concern. The ongoing exploitation and degradation of cockatoos in New Guinea, in contrast with the abundance and even pest status of some Australian populations, suggests a higher priority protection is required for the former (Ackroyd 1989; Cameron 2007; Olah et al. 2016).

Second, without accurate genetic considerations, re-wilding initiatives and translocations could have harmful consequences and need to be well-managed and carefully curated. This applies especially to cases like these cockatoos where the situation is perhaps not dire enough to warrant the need for the mixing of lineages to avoid extinction of species and/or subspecies (i.e. genetic rescue). Kwok et al. (2021) noted several organizations in Australia rehabilitating captive or injured birds for release and captive *C. galerita s.l.*, from Jakarta, Indonesia have already been rewilded into West Papua (Zein et al. 2017). At the species-level, both *C. galerita s.s.* and *C. triton* occur on New Guinea, though likely with limited overlap in distribution ranges based on bioacoustics patterns and possibly different habitat preferences—*C. galerita* now likely being restricted to the Trans-Fly ecodomain where open forest, woodlands, and savannahs dominate as opposed to denser forest types (e.g. rainforests). Introductions or translocations of the wrong species in the wrong place may result in added competition (effectively jeopardizing one species or the other) or lead to hybridization events (Baker et al. 2014). Hybridization with closely related species has been widely documented in birds (e.g. Rheindt and Edwards 2011). In rare instances, this may result in heterosis or hybrid vigor, causing population expansions threatening other native fauna (Arnold and Martin 2010; Rius and Darling 2014; Blanco-Aguiar et al. 2022; Vedder et al. 2022; Porretta and Canestrelli 2023). However, hybridization is more commonly linked to weakness of offspring due to reduced reproductive fitness, higher predation, and reduced cognitive ability (Johnsgard 1971; Casas et al. 2012; McQuillan et al. 2018; Ålund et al. 2024). Whatever the effect of hybridization, when coupled with competition, it is likely to drive major ecosystem imbalances affecting conservation efforts. Similar concerns are also echoed at the intraspecific level for ESUs of *C. galerita s.s.* (i.e. *C. g. galerita* and *C. g. fitzroyi*). Although admixture is shown between the two ESUs (Fig. 4b), the introductions of birds not conforming in genetic signature from one location to another could still negatively impact localised adaptations or drive unnatural population irruptions. Potential outbreeding depression or hybrid vigor requires investigation for this species.

Finally, given the evolution and the apparent biogeographic preference of *C. galerita* for more open forests, woodlands and savannahs and *C. triton* for denser rainforest ecosystems, the care and protection of these biomes should be at the forefront of these species' conservation efforts. Forest and woodland loss in New Guinea and Australia is accelerating due to various anthropogenic factors (Filer et al. 2009; Bradshaw 2012; Ward et al. 2019; Gamoga et al. 2021; Gaveau et al. 2021). Protection of these systems should offer opportunities for natural recovery of diminished populations.

## Conclusions and Directives for Future Research

This study provides the first phylogenomic and bioacoustic assessment of *C. galerita* and its historical biogeography. It also provides a phylogeographic blueprint based on genomic and bioacoustic data to compare evolutionary and distribution signatures of other species of Psittaciformes distributed across Australia and Melanesia. We show that *C. galerita s.l.* needs to be split into two species, namely *C. galerita* and *C. triton*. Moreover, we indicate that *C. galerita s.s.* most reliably contains two ESUs across our sampled distribution range. These ESUs broadly align with the contemporary subspecies *C. g. galerita* and *C. g. fitzroyi*, with the former extending into the Trans-Fly ecodomain of southern New Guinea. The taxonomy of *C. galerita s.l.* thus requires revision and the reassessment of distribution ranges and conservation measures. Additionally, protective legislation may need to be updated to capture the genomic and bioacoustic evidence presented herein. However, the results also open a treasure trove of questions for future research. For example, a lack of genomic sampling from New Guinea means we can make few comments about support for cryptic intraspecific diversity there or the status of other currently synonymised subspecies from that region and which may warrant elevation at least to ESU status. This is because our genomic sampling in New Guinea has been limited to two subregions across the island's South and South-East. This information is highly useful in areas like New Guinea where translocations and reintroductions are being undertaken to effectively counteract a long history of exploitation. Moreover, the restoration of *C. triton* to species-level (for at least south-eastern New Guinean cockatoos from Oro Province) would benefit from further corroboration. Sampling across more of New Guinea, and ideally including type material from museums, would be ideal. Additionally, our study failed to obtain wild samples for *C. g. eleonora* (the subspecies restricted to the Aru Islands). Hence, it remains unknown whether it may represent an ESU of *C. galerita s.s.* or not in light of the genetic evidence presented herein. Future studies could build onto this by resolving the phylogenetic and taxonomic position of *C. g. eleonora*. This is all the more urgent considering that many of the conservation challenges in New Guinea are effectively echoed in the Aru Islands and parts of Wallacea.

## Supplementary Material

msae222_Supplementary_Data

## Data Availability

Processing scripts are available from GitHub (github.com/AFSands/Genomic-and-Acoustic-Biogeography-of-the-Iconic-Sulphur-Crested-Cockatoo) while VCF files and other analytical inputs are available from the Dryad Digital Repository (doi.org/10.5061/dryad.ghx3ffbxc; Sands et al. 2024).

## Supplementary Material

Supplementary information, figures and tables may be found in Appendix A (Supplementary Information), Appendix B (Supplementary Tables) and Appendix C (Supplementary Figures) as available in Supplementary material online. Supplementary material is available at *Molecular Biology and Evolution* online.
