# Supplementary material for: Genomic and Acoustic Biogeography of the Iconic Sulphur-crested Cockatoo Clarifies Species Limits and Patterns of Intraspecific Diversity
Source: Mol Biol Evol. 2024 Oct 24;41(11):msae222. doi: 10.1093/molbev/msae222 (PMC11586666; doi:10.1093/molbev/msae222)
Supplement: msae222_Supplementary_Data [file msae222_supplementary_data.zip › AppendixASupplementaryInformation_vF5.pdf]

APPENDIX A

Supplementary Information

Genomic and Acoustic Biogeography of the Iconic Sulphur-crested Cockatoo Clarifies Species Limits and Patterns of Intraspecific Diversity

Arthur F. Sands[1], Astrid A.L. Andersson[1], Kerry Reid[1], Taylor Hains[2,3], Leo Joseph[4], Alex Drew[4], Ian J. Mason[4], Frank E. Rheindt[5], Caroline Dingle[1,6], Juha Merilä[1,7].

[1] Area of Ecology & Biodiversity, School of Biological Sciences, The University of Hong Kong, Hong Kong, Hong Kong SAR.
[2] Committee on Evolutionary Biology, University of Chicago, Chicago, USA
[3] Negaunee Integrative Research Center, Field Museum of Natural History, Chicago, USA
[4] Australian National Wildlife Collection, CSIRO National Research Collections Australia, Canberra, Australia
[5] Department of Biological Sciences, National University of Singapore, Singapore, Singapore
[6] Biology Department, Capilano University, North Vancouver, Canada
[7] Ecological Genetics Research Unit, Faculty of Biological and Environmental Sciences, University of Helsinki, Helsinki, Finland

Corresponding author: sands@hku.hk

SUPPLEMENTARY METHODS AND RESULTS

DNA extraction

DNA was extracted from the tissue or blood samples via a DNeasy Blood & Tissue Kit (QIAGEN) and following the manufacturer's recommendations with otherwise minor alterations: Our changes included extending the incubation digestion period to 24 hrs and, in some instances, eluting our final DNA extracts in 100 µl (as opposed to 200 µl) of ultrapure water or elution buffer, undertaken in two spins of 50 µl.

Reference genome selection

The reference genome of the Palm Cockatoo (*Probosciger aterrimus*, GenBank accession no. GCA_013397665.1; Feng et al. 2020) was used for the current study. The selection of this reference genome, over more closely related species, was based on: 1) The most well-constructed and reliable cockatoo reference genome available with the fewest contigs – where the quality of reference genomes over evolutionary relatedness may sometimes be preferable (Peona et al. 2020), 2) personal communication with authors/providers of other cockatoo reference genomes (Taylor Hains, personal communication), 3) the conservative structure of bird genomes (Tiersch & Wachtel 1991; Galla et al. 2018; Bravo et al. 2021), 4) the wish to not bias analyses by using one of the ingroup species and 5) the average pairwise divergence of *P. aterrimus* and *Cacatua galerita* calculated to be ~3% (Table i) and thus generally in keeping with some prescribed recommendations (e.g. Prasad et al. 2022).

Table i. Genome divergence between *P. aterrimus* (GCA_013397665.1) and *C. galerita* (GCA_035583095.1).*

Number of windows	30142
Average size of windows	20000.8bp
Average pairwise divergence	3.149%
Total sites	602865426bp

* To calculate pairwise divergence between *C. galerita* and *P. aterrimus*, we aligned the *C. galerita* genome to the *P. aterrimus* genome using LASTAL (Kiełbasa et al. 2011) and filtering the resulting MAF alignment file for one-to-one hits. The pairwise divergence was calculated by running the resulting one-to-one MAF with maffilter using a minimum block length of 10,000bp. The average block length and average pairwise divergence was then average over the total number of blocks used in the MAF alignment file to calculate a pairwise divergence between these two species. Commands are available at github.com/thainsCEB/ParrotCytogenomics.

Pseudochromosome layout of the Palm Cockatoo reference genome

The Kakapo, *Strigops habroptilus*, chromosome-level reference genome (GenBank genome accession no. GCF_004027225.2; Dussex et al. 2021) was used for scaffolding and orienting the scaffolds of the *P. aterrimus* reference genome (GenBank accession no. GCA_013397665.1; Feng et al. 2020) on the high-performance computer operated by the Grainger Bioinformatics Center at the Field Museum of Natural History. The *S. habroptilus* genome was first filtered to remove all unlocalized and unplaced scaffolds using custom scripts to remove sequence headers that followed the Vertebrate Genomes Project (VGP) naming format from a list of sequence headers and then using 'seqtk' and 'subseq' to retain only chromosome-level scaffolds. The fasta headers of the remaining chromosome-scaled scaffolds

were formatted into chromosome names following conventions applied to birds using custom scripts (https://github.com/thainsCEB/birbscripts). The *P. aterrimus* genome was then mapped to the formatted *S. habroptilus* genome using RagTag 2.1.0 (Alonge et al. 2019) into pseudochromosomes corresponding to the *S. habroptilus* karyotype.

GATK pipeline

In summary, the GATK 4.3 (McKenna et al. 2010) best practices pipeline applied herein included first creating a training dataset of hard-filtered SNPs and indels for later filtering. Here, the HaplotypeCaller function was invoked to call variants for each specimen and CombineGVCFs was used to merge outputs for all specimens into a single file. VariantFiltration on single nucleotide polymorphisms (SNPs) and indels, using recommended rigid settings (SNPs: QD < 2.0, QUAL < 30.0, SOR > 3.0, FS > 60.0, MQ < 40.0, MQRankSum < -12.5, ReadPosRankSum < -8.0; Indels: QD < 2.0, QUAL < 30.0, FS > 200.0, MQRankSum < -20.0), was then applied before Bcftools 1.16 view (Li et al. 2009) was used to select only passed variants. Secondly, the hard-filtered variants were used to, recalibrate base quality scores in bam files for each specimen with the GATK's BaseRecalibrator and ApplyBQSR, before Samtools was again used to sort and index the respective recalibrated bam files. Thirdly, HaplotypeCaller was applied to the recalibrated bam files of all specimens to call variants and merged with CombineGVCF. Finally, VariantRecalibrator and ApplyVQSR were used to recalibrate, filter and mark passed variants aided by the training dataset of hard-filtered SNPs.

More information on calibration and dating methods

<u>Selection of *Cacatua alba* as the outgroup for SNAPP dating</u>

Cacatua alba was selected as the outgroup for the dated SNAPP phylogeny based on the phylogenies of White et al. (2011) and Selvatti et al. (2022), and later Smith et al. (2023). The choice of *C. alba* over more closely related *Cacatua sulphurea* was made due to: 1) *C. sulphurea* specimens, used in the current study (Table S2), being of feral origin and thus possibly being influenced by introgression with *C. galerita* and, 2) The divergence date between *C. galerita* and *C. sulphurea* potentially being influenced by the paraphyly in *C. galerita* – we could not be confidently sure which lineage of *C. galerita* was incorporated into either White et al. (2011) or Selvatti et al. (2022) with absolute certainty. Both these factors would have jeopardised dating comparability with the existing literature.

<u>PSMC-derived calibration</u>

We calibrated our dated phylogeny with a PSMC-derived divergence date (generated with mutation rate data for Psittaciformes; Martini et al. 2021; Bergeron et al. 2023) as opposed to incorporating dates from three contemporary topologies in the literature (White et al. 2011; Selvatti et al. 2022; Smith et al. 2023). Two major reasons exist for this choice: Firstly, previous research on the divergences in *Cacatua* has mostly relied on the fossil record linked to basal divergences in Psittaciformes or those in Psittaciformes groups outside of Cacatuidae for calibration. Only a single beak fossil from Cacatuidae has been incorporated into these studies, from the early-mid Miocene (Boles 1993), which lacks modern scrutiny. Secondly, recent literature following genomic approaches suggests that divergence dates in cockatoos may be

orders of magnitude younger than previously thought (Stiller et al. 2024 *vs* White et al. 2011; Selvatti et al. 2022) and that mutation rate estimates are suggested to be more reliable in instances where the fossil records of groups are poor (Tiley et al. 2020; Pozzi & Penna 2022).

For this, we aligned and overlapped our PSMCs of individuals from Tasmania, South Australia and New South Wales with those from Tiwi Islands (Northern Territory) and Western Australia. Divergence dates between south-eastern and north-western *C. galerita* were used to calibrate the phylogeny (and the onset of diversification within *C. galerita*) – these were taken from points where PSMCs began to differentiate among the assessed individuals. We excluded PSMCs of individuals from Queensland (including the Cape York Peninsula) and the mainland Northern Territory of Australia from our consideration to avoid any potential adverse effects resulting from possible admixture (Figs 4B, S2). Divergence dates were averaged across assessed individuals to calibrate the divergence point, with the 95% highest posterior density (HPD) incorporating the maximum and minimum dates from among individual comparisons of divergence estimates (Table ii).

Table ii. Approximate divergence estimates (in thousand years ago "Kya") between selected individuals from among the PSMCs (Fig. 5) used for calibrating the dated phylogeny generated with SNAPP 1.6.1 through BEAST 2.7.5 (Fig. 6C). Where divergence dates differ substantially between the PSMC averages and bootstrap replicates, the latter is given in brackets. The minimum and maximum across comparisons are bolded and the average divergence between south-eastern and north-western *C. galerita* = 232.44 Kya.

**PSMC**	**G** (B48687; Tiwi Islands)	**H** (B50845; Western Australia)	**I** (B60554; Western Australia)
**A** (B49784; Tasmania)	202	280	200
**B** (B53845; South Australia)	215	290	**310**
**C** (B29233; New South Wales)	215	260	**120 (90)**

To ensure our PSMC-derived calibration was reliable we validated our calibration by also modelling demographic history using SMC++ (Terhorst et al. 2017; github.com/popgenmethods/smcpp) for the divergence of the same key lineages. A VCF file containing 10,106,311 autosomal SNPs for a subset of samples from Australia representing the two key lineages (i.e. *C. g. galerita*, n = 38; *C. g. fitzroyi*, n = 11; excluding any potentially admixed individuals under $K = 2$ or $K = 3$ to prevent adverse effects) was used as an original input (see Table S2 for sample subset used). The generational mutation rate and generation time for *C. galerita* remained the same as set for PSMCs and the distinguished individuals were represented by specimens containing ≥ 25× coverage. A missing cut-off was defined by calculating the maximum length among runs of homozygosity in Plink 1.90 (Chang et al. 2015) for each of the two lineages. SMC++ plots were generated for each lineage independently and also as combined to estimate splits (i.e. divergences) between lineages. Ten replicates were generated for each assessment type to ensure a level of congruence among outputs. All computational scripts and support files used for SMC++ analyses can be found on the Dryad Digital Repository (github.com/AFSands).

<u>The results of calibration date validation by SMC++</u>

The temporal trends in SMC++ effective population size (N_e) estimates between the two key lineages generally mimicked those observed in the individual PSMC plots (Fig. 5) and

importantly the divergence dates determined through SMC++ corroborated those established through PSMCs that were used for phylogenetic calibration (Table ii; Fig. i). Both methods depict the similar distinguishing greater population expansion and then contraction seen in north-western *C. g. fitzroyi* specimens as compared with those of south-eastern *C. g. galerita* over the last ~300 Ky is observed (Figs 5, i). The average among modelled SMC++ splits (237.68 Kya) aligned very closely with the mean PSMC-derived divergence date used for SNAPP phylogenetic calibration (232.44 Kya; Table ii). Moreover, the minimum (224.81 Kya) and maximum (261.52 Kya) SMC++ splits between lineages (among replicates) fall within the confidence intervals assigned for this phylogenetic dating (95% HPD = 466–90 Kya; Fig. i). It is additionally worthwhile mentioning that all SMC++-derived divergence dates are also contained within 95% HPD ultimately estimated for this node in the phylogeny (Figs 6C, i).

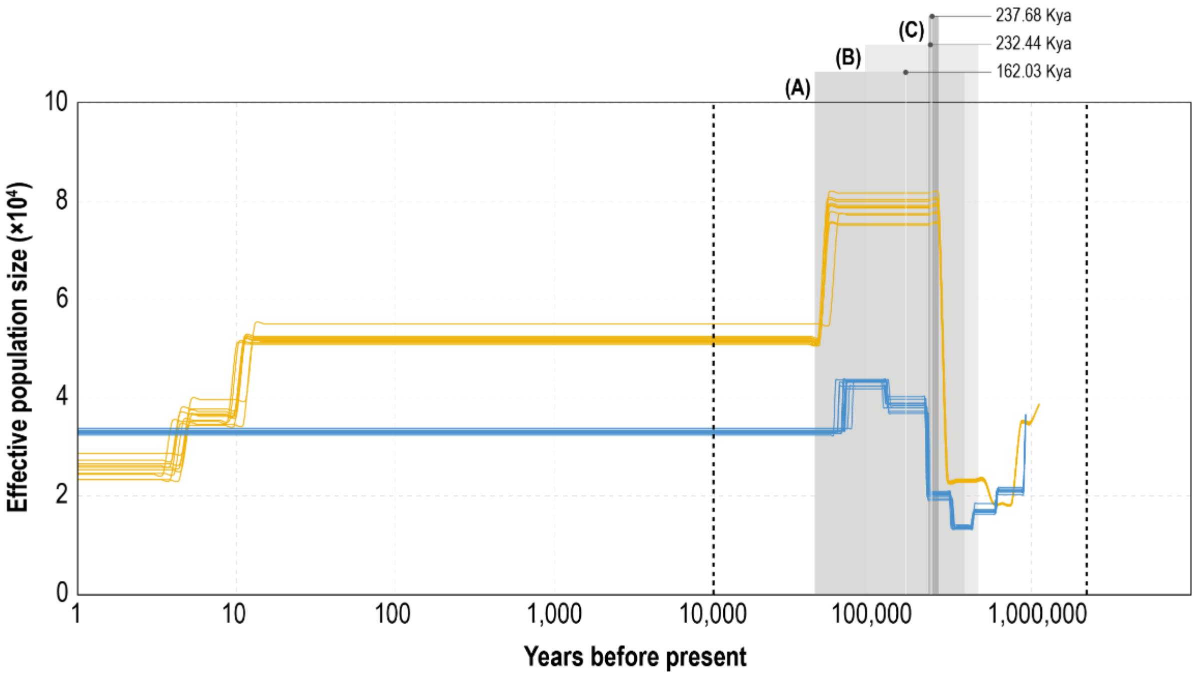

Figure i. Ten replicates of individual SMC++ temporal N_e plots generated for both key lineages (yellow = *C. g. fitzroyi*, blue = *C. g. galerita*) to validate PSMC-derived calibration dates set for phylogenetic dating of *C. galerita*. Dashed black lines in the plot area roughly enclose the temporal duration covered in the PSMCs (Fig. 5). Three transparent grey boxes depict: (A) the eventual 95% HPD interval as estimated for the calibrated node by the SNAPP phylogeny (Fig. 6C) with the median date denoted, (B) the calibration interval determined through PSMC divergences in N_e with the mean date set indicated and (C) the maximum and minimum bounds of splits modelled through SMC++ replicates for the divergence of the two lineages with the mean signified.

Caveats: PSMC-derived calibration *vs* secondary dating from the contemporary literature

We acknowledge that our phylogenetic dates are an order of magnitude younger than those in the literature (Fig. S4) and that our dates may too be worthy of scrutiny given no reliable mutation rate data exists directly for *Cacatua* as yet (Zhang et al. 2023). However, our divergence dates align well with expected responses to climatic and environmental changes (see Discussion) and the mutation rate used comes from an average of Psittaciformes with similar life history characteristics and generation times (Martini et al. 2021; Bergeron et al. 2023). Moreover, the younger divergence time between the *Cacatua* spp. may also better

explain the ability to naturally hybridize (Ford 1985; Kentish & Brennan 2004; Rowley et al. 2020; Rowley & Boesman 2020; Hingston 2022) – even in some instances with possible viable offspring.

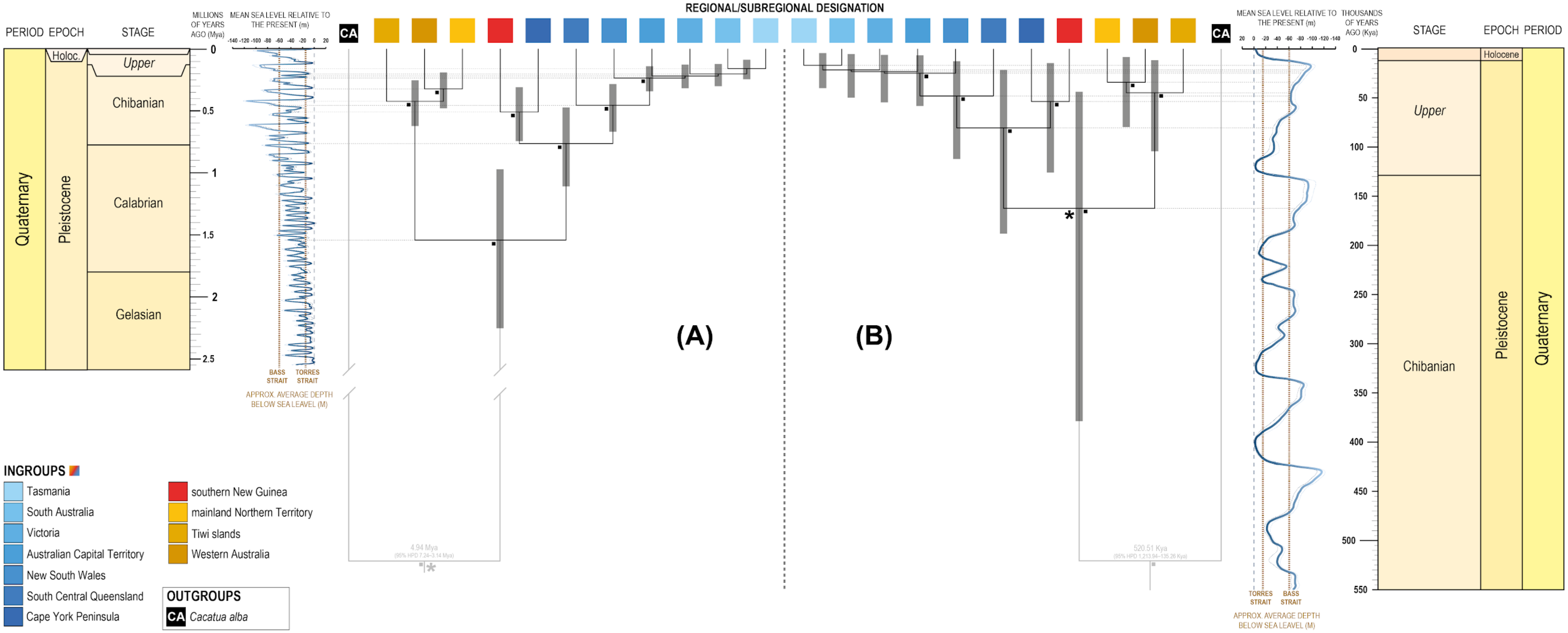

Figure ii. Dated phylogenies, generated with SNAPP 1.6.1 through BEAST 2.7.5, following (A) conventional dates from the literature *vs* (B) PSMC-derived divergence dates, generated with BEAST 2.7.5, using 34 specimens and 10,000 random SNPs. The colour of tip blocks corresponds with the key. Small black squares and grey bars at nodes reflect significant posterior probabilities (PP) and 95% confidence intervals around divergence dates respectively. Dates are aligned to Pleistocene sea levels (Berends et al. 2020) and a relative geological time scale in both instances. The portion of the phylogeny not incorporating *C. galerita* intraspecific diversity has been shaded lighter. Colour blocks at tips reflect the origin of individuals incorporated therein as denoted in the key.

Isolation-by-distance

Pairwise genetic distance matrixes for *C. galerita* intraspecific diversity (n = 88; excluding interspecific *C. g. triton* Oro), as calculated through ngsDist (Vieira et al. 2015) with the intext mentioned VCF file for 100 bootstrap replicates. Here pairwise comparisons among replicates were averaged in R 4.2.2 (R Core Team 2022) to generate a consensus matrix for genetic difference. Additionally, for geographic distance; since longitude, latitude and geographical distance among localities were correlated, we only included geographical distance in the distance-based redundancy analysis (db-RDA). Principal coordinates analysis was performed on the matrix of pairwise geographical distances and the first principal component was used as a single continuous variable in the db-RDA (Grant 2015). Below follow the results from this analysis (Tables iii–vii):

Table iii. Correlation between latitude, longitude and geographic distance (GGD).

	Latitude	Longitude	GGD
Latitude	1	-0.6154721	0.9859366
Longitude	-0.6154721	1	-0.7382507
GGD	0.9859366	-0.7382507	1

Table iv. Results of db-RDA for the dataset (genetic distance (GD) *vs*. GGD).

	Inertia	Proportion	Rank
Total	1.4495	1.0000	
Constrained	0.1269	0.0875	1
Unconstrained	1.3226	0.9125	86

Table v. Eigenvalues for constrained unconstrained axes.

	Axes	Eigenvalues
Constrained axes	CAP1	0.12688
Unconstrained axes (first 10 of 86)	MDS1	0.08571
	MSD2	0.02990
	MDS3	0.02632
	MDS4	0.02326
	MDS5	0.02219
	MDS6	0.02046
	MDS7	0.01953
	MDS8	0.01934
	MDS9	0.01894
	MDS10	0.01802

General scaling constant of scores: 3.351064

Table vi. Results of the permutation test for capscale under reduced model, where permutations were free and the number of permutations = 9999.

	Df	Variance	F	Pr(>F)
Model	1	0.12688	8.25	0.0001
Residual	86	1.32260		

Table vii. R^2 and adjusted R^2 values for the dataset (GD *vs.* GGD).

	Coefficient of determination
R^2	0.08753301
adj. R^2	0.07692292

SUPPLEMENTARY DISCUSSION

Lack of support for the reinstatement of long synonymised subspecies – most especially across Australia.

We found limited support for cryptic diversity across the sampled range warranting reinstatement of long synonymized subspecies or ESUs (Figs 3, 6B, 6C, 6E; Table S1). For example, *C. g. melvillensis* (Mathews, 1912), *C. g. licmetorhyncha* (Bonaparte, 1850) or *C. g. interjecta* (Mathews, 1917), described from the Tiwi Islands, Tasmania and Victoria respectively, could all be argued to be monophyletic in our analyses. If reinstated, however, they would cause polyphyly in *C. g. galerita* and *C. g. fitzroyi* respectively and necessitate potentially naming several new subspecies that would make little morphological, acoustic or geographic sense. Furthermore, they would not correspond well with ESU delimitations we have suggested. Other subspecies long synonymized by most authors, such as *C. g. queenslandica* from Cape York Peninsula, Australia are not always monophyletic (Fig. 3 *vs* Fig. 6C). Similarly, while *C. g. triton* TF is in some analyses monophyletic and sister to *C. g. galerita*, the levels of genomic and acoustic differentiation are extremely shallow and it is difficult to distinguish it from specimens of *C. g. galerita* from Cape York Peninsula (Figs 2, 3, 6C, 6E).

These results are not unexpected: Firstly, subsequent workers have usually determined that the differentiating traits claimed for these populations were spurious or questionable, hence the synonymisation of many of these subspecies (e.g. Mathews 1927; Peters 1937; Forshaw 1969). Secondly, several subspecies have been described from nearshore islands (e.g. see Mathews 1917), for example *C. g. melvillensis* and *C. g. licmetorhyncha* as mentioned above. Herein, glacial cycles caused dramatic fluctuations in sea levels (Fig. 6A, 6C). These fluctuations have been widely noted to have caused repeated connections and disconnections of surrounding islands to continental Australia, including New Guinea, Aru Islands, Tiwi Islands, Kangaroo Island, Tasmania and some of the Milne Bay islands of Papua New Guinea, from each of which *C. galerita* subspecies have been named. The connections of these islands to mainland Australia and New Guinea have occurred several times over the last million years and at least twice (for prolonged periods) in the last two hundred thousand years – most recently ceasing at the end of the LGP ~12 Kya (Allen et al. 2020). Moreover, low-lying coastal areas of many of these islands may have even possibly disappeared during interglacial highstands, greatly reducing and/or shifting habitat and the ability of these islands to sustain viable populations

(Allen et al. 2020; Berends et al. 2020). The temporal isolation or persistence of populations on these islands may not have been sustained long enough for the evolution of differentiation warranting recognition as subspecies/ESUs. Further, any differentiation generated during preceding interglacial isolations may have been eroded when land bridges existed. Indeed, our ancestral area estimations suggest that the islands where *C. galerita* was sampled for this study, show only very recent indications of colonisation (e.g. see Tasmania, Tiwi Islands and southern New Guinea; Fig. 6C). Finally, even if some may consider *C. galerita s.l.* to have poor long-distance flight capabilities, the distances between some islands and continental populations are probably not beyond the birds' dispersal capabilities to cross in exceptional circumstances, as has been documented in other similar sized Psittaciformes (e.g. New Zealand Kākā, Diamond 1984; Beauchamp et al. 2009; Forshaw & Knight 2017). The Tiwi Islands, for example, are separated from continental Australia by the Clarence and Dundas straits. However, several islets in the former make the maximum overwater extent of only about ~12.5 km. Moreover, founder populations, such as those on islands reached by colonising individuals, are likely to be small and phenotypic differences, if any, may more likely be driven by population size bottleneck effects as well as by variation in local environmental conditions (Palkovacs 2003; also see Le Gros et al. 2016).

SUPPLEMENTARY ACKNOWLEDGEMENTS

We would like to thank the Grainger Bioinformatics Center at the Field Museum of Natural History for access to high-performance computing facilities for the generation of the pseudo-chromosome layout of the Palm Cockatoo reference genome.

SUPPLEMENTARY REFERENCES

Allen JR, Forrest M, Hickler T, Singarayer JS, Valdes PJ, Huntley B. 2020. Global vegetation patterns of the past 140,000 years. *J. Biogeogr.* 47(10):2073–2090.

Alonge M, Soyk S, Ramakrishnan S, Wang X, Goodwin S, Sedlazeck FJ, Lippman ZB, Schatz MC. 2019. RaGOO: Fast and accurate reference-guided scaffolding of draft genomes. *Genome Biol.* 20:224.

Beauchamp A, Hansen K, Pilon G. 2009. Canopy and above canopy movements of birds on Whatupuke Island, New Zealand. *Notornis* 56:213–216.

Berends CJ, De Boer B, Van De Wal RS. 2021. Reconstructing the evolution of ice sheets, sea level and atmospheric CO_2 during the past 3.6 million years. *Clim. Past* 17(1):361–377.

Bergeron LA, Besenbacher S, Zheng J, Li P, Bertelsen MF, Quintard B, Hoffman JI, Li Z, St. Leger J, Shao C, Stiller J. 2023. Evolution of the germline mutation rate across vertebrates. *Nature* 615(7951):285–291.

Boles WE. 1993. A new cockatoo (Psittaciformes: Cacatuidae) from the Tertiary of Riversleigh, northwestern Queensland, and an evaluation of rostral characters in the systematics of parrots. *Ibis* 135:8–18.

Bonaparte CL. 1850. Nouvelles espèces ornithologiques. *C. r. hebd. séances Acad. sci.* 30:131–139.

Bravo GA, Schmitt CJ, Edwards SV. 2021. What have we learned from the first 500 avian genomes? *Annu. Rev. Ecol. Evol. S.* 52:611–639.

Chang CC, Chow CC, Tellier LC, Vattikuti S, Purcell SM, Lee JJ. 2015. Second-generation PLINK: Rising to the challenge of larger and richer datasets. *Gigascience* 4(1):s13742-015.

Diamond JM. 1984. Distributions of New Zealand birds on real and virtual islands. *New Zeal. J. Ecol.* 7:37–55.

Dussex N, Van Der Valk T, Morales HE, Wheat CW, Díez-del-Molino D, Von Seth J, Foster Y, Kutschera VE, Guschanski K, Rhie A, et al. 2021. Population genomics of the critically endangered kākāpō. *Cell Genom.* 1(1):100002.

Feng S, Stiller J, Deng Y, Armstrong J, Fang QI, Reeve AH, Xie D, Chen G, Guo C, Faircloth BC, et al. 2020. Dense sampling of bird diversity increases power of comparative genomics. *Nature* 587(7833):252–257.

Ford J. 1985. Species limits and phylogenetic relationships in corellas of the *Cacatua pastinator* complex. *Emu* 85(3):163–180.

Forshaw JM. 1969. Australian Parrots. Melbourne (AUS): Lansdowne Press. pp. 306.

Forshaw JM, Knight F. 2017. Vanished and vanishing parrots: Profiling extinct and endangered species. Clayton South (AUS): CSIRO Publishing. pp. 322.

Galla SJ, Forsdick NJ, Brown L, Hoeppner MP, Knapp M, Maloney RF, Moraga R, Santure AW, Steeves TE. 2019. Reference genomes from distantly related species can be used for discovery of single nucleotide polymorphisms to inform conservation management. *Genes* 10(1):9.

Grant WS. 2015. Problems and cautions with sequence mismatch analysis and Bayesian skyline plots to infer historical demography. *J. Hered.* 106(4):333–346.

Kentish B, Brennan D. 2004. Wild-caught Long-billed Corella '*Cacatua tenuirostris*' × Sulphur-crested Cockatoo '*C. galerita*' hybrids from western Victoria. *Aust. Field Ornithol.* 21(2):76–78.

Kiełbasa SM, Wan R, Sato K, Horton P, Frith MC. 2011. Adaptive seeds tame genomic sequence comparison. *Genome Res.* 21(3):487–493.

Le Gros A, Samadi S, Zuccon D, Cornette R, Braun MP, Senar JC, Clergeau P. 2016. Rapid morphological changes, admixture and invasive success in populations of Ring-necked Parakeets (*Psittacula krameri*) established in Europe. *Biol. Invasions* 18:1581–1598.

Li H, Handsaker B, Wysoker A, Fennell T, Ruan J, Homer N, Marth G, Abecasis G, Durbin R, 1000 Genome Project Data Processing Subgroup. 2009. The sequence alignment/map format and SAMtools. *Bioinformatics* 25(16):2078–2079.

Martini D, Dussex N, Robertson BC, Gemmell NJ, Knapp M. 2021. Evolution of the "world's only alpine parrot": Genomic adaptation or phenotypic plasticity, behaviour and ecology? *Mol. Ecol.* 30(23):6370–6386.

Mathews GM. 1912. A reference list to the birds of Australia. *Novitates Zoologicae* 18:171–455.

Mathews GM. 1917. The Birds of Australia. Vol. 6, pts 2. London (UK): Witherby & Co. p. 105–516.

Mathews GM. 1927. Systema Avium Australasianarum: A systematic checklist of the birds of the Australian region. London (UK): British Ornithologists Union. pp. 1047.

McKenna A, Hanna M, Banks E, Sivachenko A, Cibulskis K, Kernytsky A, Garimella K, Altshuler D, Gabriel S, Daly M, et al. 2010. The Genome Analysis Toolkit: A MapReduce framework for analyzing next-generation DNA sequencing data. *Genome Res.* 20(9):1297–1303.

Palkovacs EP. 2003. Explaining adaptive shifts in body size on islands: A life history approach. *Oikos* 103(1):37–44.

Prasad A, Lorenzen ED, Westbury MV. 2022. Evaluating the role of reference-genome phylogenetic distance on evolutionary inference. *Mol. Ecol. Res.* 22(1):45–55.

Peona V, Blom MP, Xu L, Burri R, Sullivan S, Bunikis I, Liachko I, Haryoko T, Jønsson KA, Zhou Q, et al. 2021. Identifying the causes and consequences of assembly gaps using a multiplatform genome assembly of a bird-of-paradise. *Mol. Ecol. Res.* 21(1):263–286.

Peters JL. 1937. Checklist of Birds of the World. Vol. 3. Cambridge, Massachusetts (USA): Harvard University Press. pp. 311.

Pozzi L, Penna A. 2022. Rocks and clocks revised: New promises and challenges in dating the primate tree of life. *Evol. Anthropol.* 31(3):138–153.

R Core Team. 2022. R: A language and environment for statistical computing. R Foundation for Statistical Computing, Vienna, Austria. Available from: https://www.R-project.org. Accessed November 17, 2023.

Rowley I, Boesman PFD. 2020. White Cockatoo (*Cacatua alba*), version 1.0. In: del Hoyo J, Elliott A, Sargatal J, Christie DA, de Juana E, editors. Birds of The World. Ithaca (USA): Cornell Lab of Ornithology. Available from:https://birdsoftheworld.org/bow/species/whicoc1/cur/introduction. Accessed March 18, 2024.

Rowley I, Kirwan GM, Boesman PFD. 2020. Sulphur-crested Cockatoo (*Cacatua galerita*), version 1.0. In: del Hoyo J, Elliott A, Sargatal J, Christie DA, de Juana E, editors. Birds of The World. Ithaca (USA): Cornell Lab of Ornithology. Available from: https://birdsoftheworld.org/bow/species/succoc/cur/introduction. Accessed March 18, 2024.

Selvatti AP, Galvão A, Mayr G, Miyaki CY, Russo CADM. 2022. Southern hemisphere tectonics in the Cenozoic shaped the pantropical distribution of parrots and passerines. *J. Biogeog.* 49(10):1753–1766.

Smith BT, Merwin J, Provost KL, Brumfield RT, Ferreira M, Mauck III, WM, Moyle RG, Wright T, Joseph L. 2023. Phylogenomic analysis of the parrots of the world distinguishes artifactual from biological sources of gene tree discordance. *Syst. Biol.* 72(1):221–248.

Stiller J, Feng S, Chowdhury AA, Rivas-González I, Duchêne DA, Fang Q, Deng Y, Kozlov A, Stamatakis A, Claramunt S, et al. 2024. Complexity of avian evolution revealed by family-level genomes. *Nature* published online:s41586-024-07323-1.

Terhorst J, Kamm JA, Song YS. 2017. Robust and scalable inference of population history from hundreds of unphased whole genomes. *Nat. Genet.* 49(2):303–309.

Tiley GP, Poelstra JW, Dos Reis M, Yang Z, Yoder AD. 2020. Molecular clocks without rocks: New solutions for old problems. *Trends Genet*. 36(11):845–856.

Tiersch TR, Wachtel SS. 1991. On the evolution of genome size of birds. *J. Hered.* 82(5):363–368.

Vieira FG, Lassalle F, Korneliussen TS, Fumagalli M. 2016. Improving the estimation of genetic distances from Next-Generation Sequencing data. *Biol. J. Linn. Soc.* 117(1):139–149.

White NE, Phillips MJ, Gilbert MTP, Alfaro-Núñez A, Willerslev E, Mawson PR, Spencer PB, Bunce M. 2011. The evolutionary history of cockatoos (Aves: Psittaciformes: Cacatuidae). *Mol. Phylogenet. Evol.* 59(3):615–622.

Zhang C, Reid K, Sands AF, Fraimout A, Schierup MH, Merilä J. 2023. *De novo* mutation rates in sticklebacks. *Mol. Biol. Evol.* 40(9):msad192.
