# Supplementary material for: Genomic and Acoustic Biogeography of the Iconic Sulphur-crested Cockatoo Clarifies Species Limits and Patterns of Intraspecific Diversity
Source: Mol Biol Evol. 2024 Oct 24;41(11):msae222. doi: 10.1093/molbev/msae222 (PMC11586666; doi:10.1093/molbev/msae222)
Supplement: msae222_Supplementary_Data [file msae222_supplementary_data.zip › AppendixCSupplementaryFiguresvF5.pdf]

APPENDIX C

Supplementary Figures

Genomic and Acoustic Biogeography of the Iconic Sulphur-crested Cockatoo Clarifies Species Limits and Patterns of Intraspecific Diversity

Arthur F. Sands[1], Astrid A.L. Andersson[1], Kerry Reid[1], Taylor Hains[2,3], Leo Joseph[4], Alex Drew[4], Ian J. Mason[4], Frank E. Rheindt[5], Caroline Dingle[1,6], Juha Merilä[1,7].

[1] Area of Ecology & Biodiversity, School of Biological Sciences, The University of Hong Kong, Hong Kong, Hong Kong SAR.
[2] Committee on Evolutionary Biology, University of Chicago, Chicago, USA
[3] Negaunee Integrative Research Center, Field Museum of Natural History, Chicago, USA
[4] Australian National Wildlife Collection, CSIRO National Research Collections Australia, Canberra, Australia
[5] Department of Biological Sciences, National University of Singapore, Singapore, Singapore
[6] Biology Department, Capilano University, North Vancouver, Canada
[7] Ecological Genetics Research Unit, Faculty of Biological and Environmental Sciences, University of Helsinki, Helsinki, Finland

Corresponding author: sands@hku.hk

SUPPLEMENTARY FIGURE CAPTIONS

Figure S1. *Cacatua* interspecific principal component analysis (PCA) containing *C. galerita* subspecies and outgroups *C. sulphurea* and *C. alba* (n=99). The PCA is plotted in 2D and 3D space, as calculated through Plink 2 using 2,221,243 SNPs. The 2D PCA is based on the first two principal components, while the 3D version (placed in the top left quadrant of the 2D PCA) covers the first three principal components. Circles or dots coloured in blue, red and yellow represent *C. galerita* intraspecific diversity, while black dots bordered in different colour schemes are linked to outgroups or interspecific diversity (as seen in Fig. 3): Blue dots correspond to specimens attributed to *C. g. galerita* across eastern Australia and Tasmania; red dots correspond to specimens attributed to *C. g. triton* TF from southern New Guinea; yellow dots correspond to *C. g. fitzroyi* from northern and north-western Australia; black dots encircled in green correspond to feral *C. sulphurea* from Hong Kong; black dots encircled in red correspond to specimens attributed to *C. g. triton* Oro from south-eastern New Guinea; black dots encircled in purple correspond to captive *C. alba* from Hong Kong. See text for suffixes TF and Oro used with *C. g. triton*.

Figure S2. Admixture plots assuming 2–6 genetic clusters (*K*) for *C. galerita* intraspecific diversity (n = 88) with 1,745,807 SNPs, as calculated with ADMIXTURE 1.3. Plots *K* = 2–6 have been placed on top of each other with the voucher numbers noted under the final plot (*K* = 6). Location names and coloured lines under the voucher numbers indicate the regional/subregional sampling localities (also see Fig. 1). Blue, red and yellow rectangles dividing the plots indicate specimens attributed to *C. g. galerita* (blue), *C. g. triton* TF (red) and *C. g. fitzroyi* (yellow). Genetic clusters have been coloured in grayscale in each plot with variations depending on the specific number of *K* being assessed. Bars for each specimen denote the relative genetic association of that individual belonging to a specific cluster in each scenario as a percentage. Cross-validation (c.v.) errors and the frequency of the overall admixture plot observed across ten replicates are noted in the upper left portion of each plot. Where the most frequent plot shape among the ten replicates did not match the plot with the lowest c.v. error, both plots were depicted (see *K* = 4). See text for suffixes TF used with *C. g. triton*.

Figure S3. Supporting outputs from evolutionarily significant unit (ESU) delimitation regimes. (A) Bayesian implementation of the Poisson tree processes (bPTP) with the outgroup omitted, (B) bPTP with the outgroup included, (C) Generalised Mixed Yule Coalescent (GMYC) under a single threshold and, (D) GMYC under multiple thresholds.

SUPPLEMENTARY FIGURES

Figure S1

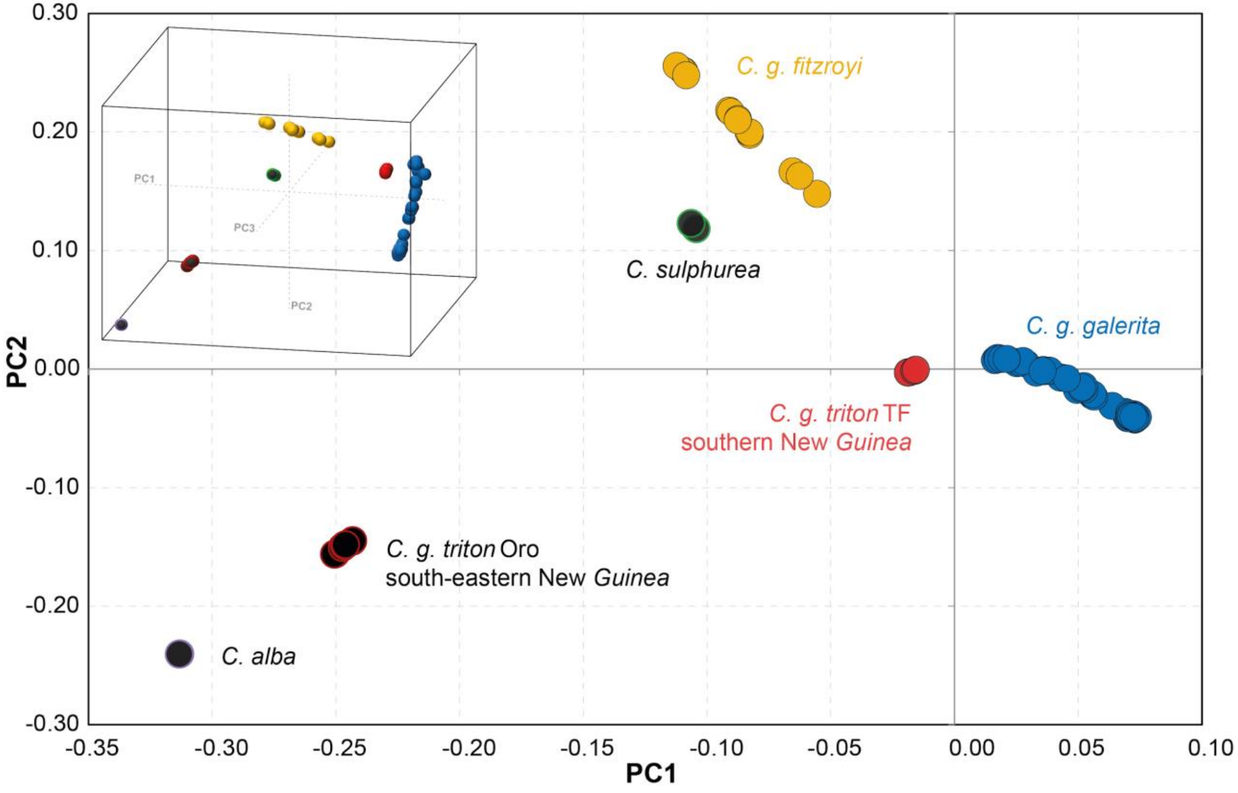

Figure S2

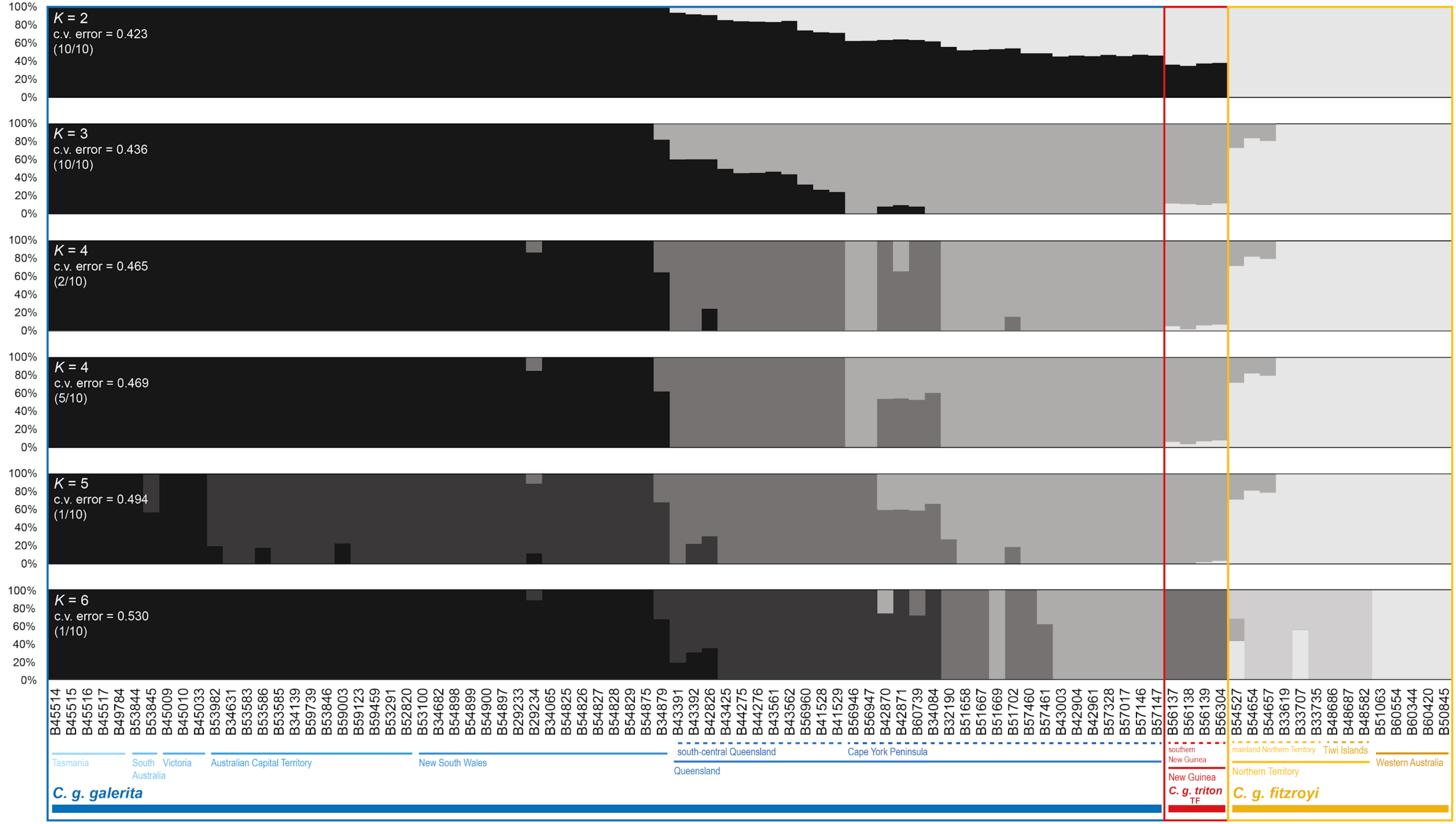

100%
80%
60%
40%
20%
0%
K = 2
c.v. error = 0.423
(10/10)
K = 3
c.v. error = 0.436
(10/10)
K = 4
c.v. error = 0.465
(2/10)
K = 4
c.v. error = 0.469
(5/10)
K = 5
c.v. error = 0.494
(1/10)
K = 6
c.v. error = 0.530
(1/10)
B45514 B45515 B45516 B45517 B49784 B53844 B53845 B45009 B45010 B45033 B53982 B34631 B53583 B53586 B53585 B34139 B59739 B53846 B59003 B59123 B59459 B53291 B52820 B53100 B34682 B54898 B54899 B54900 B54897 B29233 B29234 B34065 B54825 B54826 B54827 B54828 B54829 B54875 B34879 B43391 B43392 B42826 B43425 B44275 B44276 B43561 B43562 B56960 B41528 B41529 B56946 B56947 B42870 B42871 B60739 B34084 B32190 B51658 B51667 B51669 B51702 B57460 B57461 B43003 B42904 B42961 B57328 B57017 B57146 B57147 B56137 B56138 B56139 B56304 B54527 B54654 B54657 B33619 B33707 B33735 B48686 B48687 B48582 B51063 B60554 B60344 B60420 B50845
Tasmania
South Australia
Victoria
Australian Capital Territory
New South Wales
south-central Queensland
Cape York Peninsula
Queensland
southern New Guinea
New Guinea
mainland Northern Territory
Tiwi Islands
Northern Territory
Western Australia
C. g. galerita
C. g. triton
TF
C. g. fitzroyi

Figure S3

(A) bPTP WITH OUTGROUPS OMMITED

MAXIMUM LIKELIHOOD SOLOUTION

HIGHEST BAYESIAN SUPPORTED SOLUTION

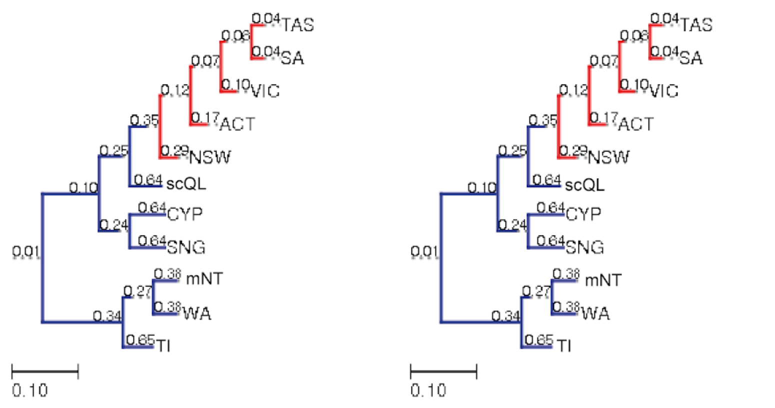

Max likilhood partition
Species 1 (support = 0.644): CYP
Species 2 (support = 0.644): sNG
Species 3 (support = 0.352): TAS, SA, VIC, ACT, NSW
Species 4 (support = 0.639): scQL
Species 5 (support = 0.650): TI
Species 6 (support = 0.376): mNT
Species 7 (support = 0.376): WA

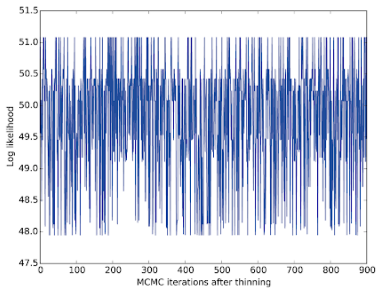

Most supported partition found by simple heuristic search
Species 1 (support = 0.352): TAS, SA, VIC, ACT, NSW
Species 2 (support = 0.639): scQL
Species 3 (support = 0.650): TI
Species 4 (support = 0.376): mNT
Species 5 (support = 0.376): WA
Species 6 (support = 0.644): CYP
Species 7 (support = 0.644): sNG

Delimitation summary
TI: 0.650
sNG: 0.644
CYP: 0.644
scQL: 0.639
mNT: 0.376
WA: 0.376
TAS, SA, VIC, ACT, NSW: 0.352
mNT, WA, TI: 0.339
NSW: 0.287
mNT, WA: 0.274
TAS, SA, VIC, ACT, NSW, scQL: 0.246
CYP, sNG: 0.242
ACT: 0.166
TAS, SA, VIC, ACT: 0.121
TAS, SA, VIC, ACT, NSW, scQL, CYP, sNG: 0.103
VIC: 0.099
TAS, SA, VIC: 0.068
TAS, SA: 0.055
TAS: 0.043
SA: 0.043
TAS, SA, VIC, ACT, NSW, scQL, CYP, sNG, mNT, WA, TI: 0.011

Acceptance rate = 0.629
Merge = 49373
Split = 49979
Estimated number of ESUs is between 1 and 7
Mean number of ESUs = 5.78

(B) bPTP WITH OUTGROUPS INCLUDED

MAXIMUM LIKELIHOOD SOLOUTION

HIGHEST BAYESIAN SUPPORTED SOLUTION

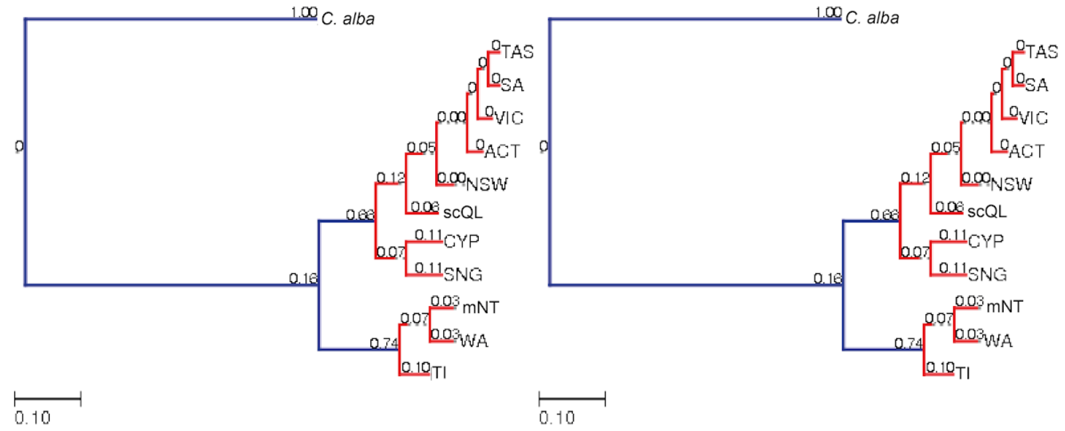

Max likilhood partition
Species 1 (support = 1.000): *C. alba*
Species 2 (support = 0.665): TAS, SA, VIC, ACT, NSW, scQL, CYP, sNG
Species 3 (support = 0.744): mNT, WA, TI

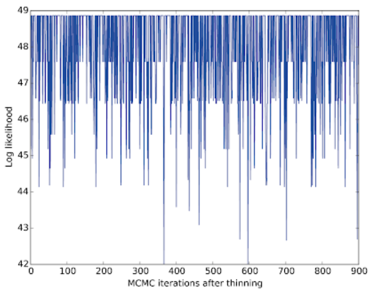

Most supported partition found by simple heuristic search
Species 1 (support = 1.000): *C. alba*
Species 2 (support = 0.665): TAS, SA, VIC, ACT, NSW, scQL, CYP, sNG
Species 3 (support = 0.744): mNT, WA, TI

Delimitation summary
C. alba: 1.000
mNT, WA, TI: 0.744
TAS, SA, VIC, ACT, NSW, scQL, CYP, sNG: 0.665
TAS, SA, VIC, ACT, NSW, scQL, CYP, sNG, mNT, WA, TI: 0.155
TAS, SA, VIC, ACT, NSW, scQL: 0.123
sNG: 0.105
CYP: 0.105
TI: 0.101
CYP, sNG: 0.074
mNT, WA: 0.071
scQL: 0.057
TAS, SA, VIC, ACT, NSW: 0.052
mNT: 0.030
WA: 0.030
NSW: 0.004
TAS, SA, VIC, ACT: 0.004

Acceptance rate = 0.375
Merge = 49977
Split = 50023
Estimated number of ESUs is between 2 and 8
Mean number of ESUs = 3.32

(C) GMYC: SINGLE THRESHOLD

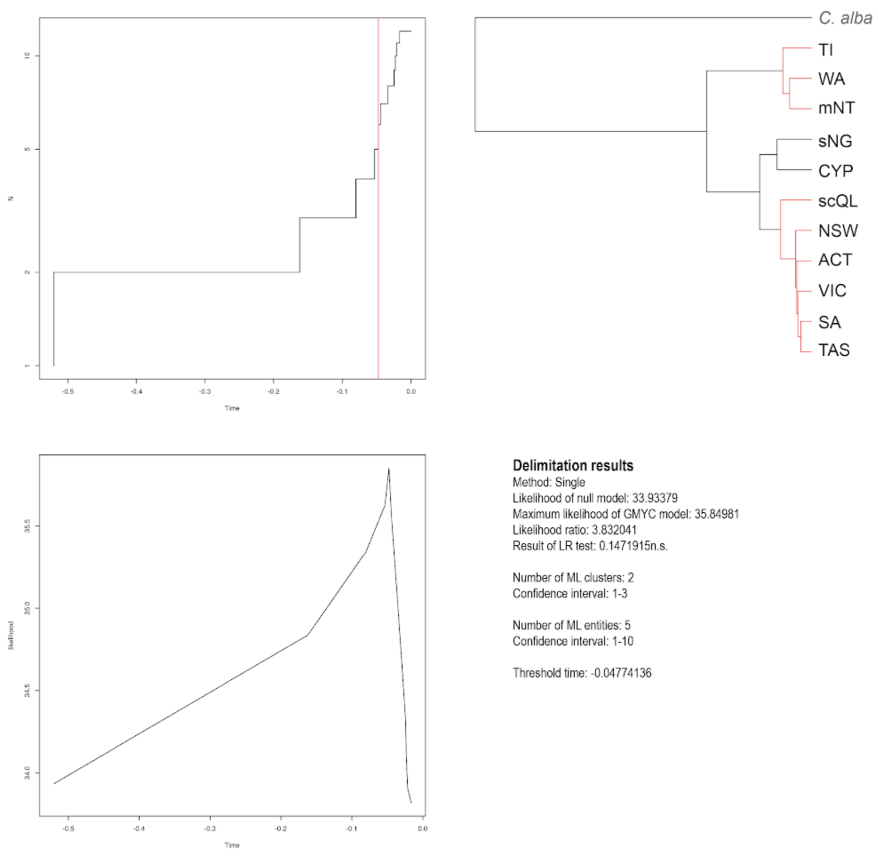

Delimitation results
Method: Single
Likelihood of null model: 33.93379
Maximum likelihood of GMYC model: 35.84981
Likelihood ratio: 3.832041
Result of LR test: 0.1471915n.s.

Number of ML clusters: 2
Confidence interval: 1-3

Number of ML entities: 5
Confidence interval: 1-10

Threshold time: -0.04774136

(D) GMYC: MULTIPLE THRESHOLD

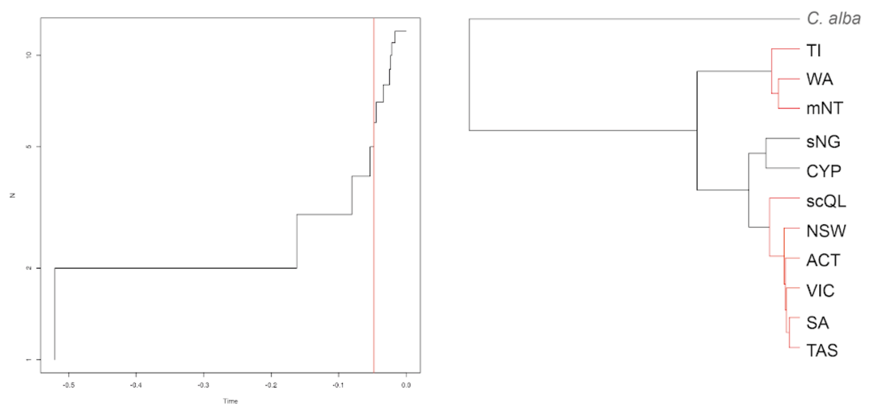

Delimitation results
Method: Multiple
Likelihood of null model: 33.93379
Maximum likelihood of GMYC model: 35.84981
Likelihood ratio: 3.832041
Result of LR test: 0.1471915n.s.

Number of ML clusters: 2
Confidence interval: 1-3

Number of ML entities: 5
Confidence interval: 1-6

Threshold time: -0.04774136
